# *Flashfm-ivis*: interactive visualization for fine-mapping of multiple quantitative traits

**DOI:** 10.1093/bioinformatics/btac453

**Published:** 2022-07-06

**Authors:** Feng Zhou, Adam S. Butterworth, Jennifer L. Asimit

**Affiliations:** 1 MRC Biostatistics Unit, University of Cambridge, Cambridge CB2 0SR, UK; 2 British Heart Foundation, Cardiovascular Epidemiology Unit, Department of Public Health and Primary Care, University of Cambridge, Cambridge CB1 8RN, UK; 3 National Institute for Health Research Blood and Transplant Research Unit in Donor Health and Genomics University of Cambridge, Cambridge CB1 8RN, UK; 4 British Heart Foundation Centre of Research Excellence, University of Cambridge, Cambridge CB1 8RN, UK; 5 Health Data Research UK Cambridge, Wellcome Genome Campus and University of Cambridge, Cambridge CB10 1SA, UK

## Abstract

**Summary:**

*flashfm-ivis* provides a suite of interactive visualization plots to view potential causal genetic variants that underlie associations that are shared or distinct between multiple quantitative traits and compares results between single- and multi-trait fine-mapping. Unique features include network diagrams that show joint effects between variants for each trait and regional association plots that integrate fine-mapping results, all with user-controlled zoom features for an interactive exploration of potential causal variants across traits.

**Availability and implementation:**

*flashfm-ivis* is an open-source software under the MIT license. It is available as an interactive web-based tool (http://shiny.mrc-bsu.cam.ac.uk/apps/flashfm-ivis/) and as an R package. Code and documentation are available at https://github.com/fz-cambridge/fiashfm-ivis and https://zenodo.org/record/6376244#.YjnarC-l2X0. Additional features can be downloaded as standalone R libraries to encourage reuse.

## 1 Introduction

Genome-wide association studies (GWAS) have successfully identified many genetic variants that are associated with diseases and traits (Claussnitzer *et al*., 2020). Identifying the causal variants that underlie genetic associations is key to translating these findings into new therapeutic targets or revealing new biological insights for diseases. Statistical fine-mapping aids this by identifying potential causal variants with the aim of reducing the number of genetic variants for follow-up in downstream functional validation experiments (Hutchinson *et al*., 2020; Spain and Barrett, 2015). As biologically related traits often have shared causal variants, multi-trait fine-mapping that shares information between traits can improve precision over single-trait fine-mapping of each trait independently (Hernandez *et al*., 2021).

There are few multi-trait fine-mapping methods that allow multiple causal variants at a single genomic region due to the computational complexity of many possible model combinations between traits. One approach is to restrict traits to have the same causal variants and allow for different effect sizes, as in fastPAINTOR (Kichaev *et al*., 2017). In contrast, flashfm (Hernandez *et al*., 2021) makes no such restrictions and uses a Bayesian framework that upweights joint models with shared causal variants. In extensive simulation comparisons, flashfm was shown to have higher precision than fastPAINTOR.

Bioinformatics tools are moving in the direction of dynamic interaction between GWAS data and plots (Supplementary Table S1), but most require some programming knowledge; none of them help to explore fine-mapping results. Non-interactive fine-mapping visualization tools include PAINTOR-CANVIS for visualizing a single set of fine-mapping results [and linkage disequilibrium (LD) structure] from PAINTOR (Kichaev *et al*., 2017) and echolocatoR (Schilder *et al*., 2022) for single-trait fine-mapping results from several methods; both require some programming knowledge.

*Flashfm-ivis* provides interactive exploration and publication-ready plots to summarize fine-mapping results from multiple traits. Linked from *flashfm-ivis,* users may use *finemap-ivis* to interact with and plot single-trait results. Table 1 compares the key features of the tools that are most similar to *flashfm-ivis*.

Flashfm multi-trait fine-mapping uses single-trait fine-mapping results from FINEMAP (Benner *et al*., 2016) or JAM (Newcombe *et al*., 2016) and we refer to either of these methods as ‘fm’. As in JAM and FINEMAP, for each trait, flashfm outputs a model posterior probability (PP) for each configuration of variants being joint causal variants for the trait. For ease of interpretation, variant groups are constructed in flashfm for both single- and multi-trait fine-mapping approaches such that variants in the same group can be viewed as exchangeable, that is, they are in high LD and rarely appear in the same model together. A 99% credible set is constructed by including the variants that appear in the top models with PPs that sum to at least 99%. Therefore, for each trait the 99% credible set has a probability of 99% of containing all the causal variants.

**Fig. 1:**
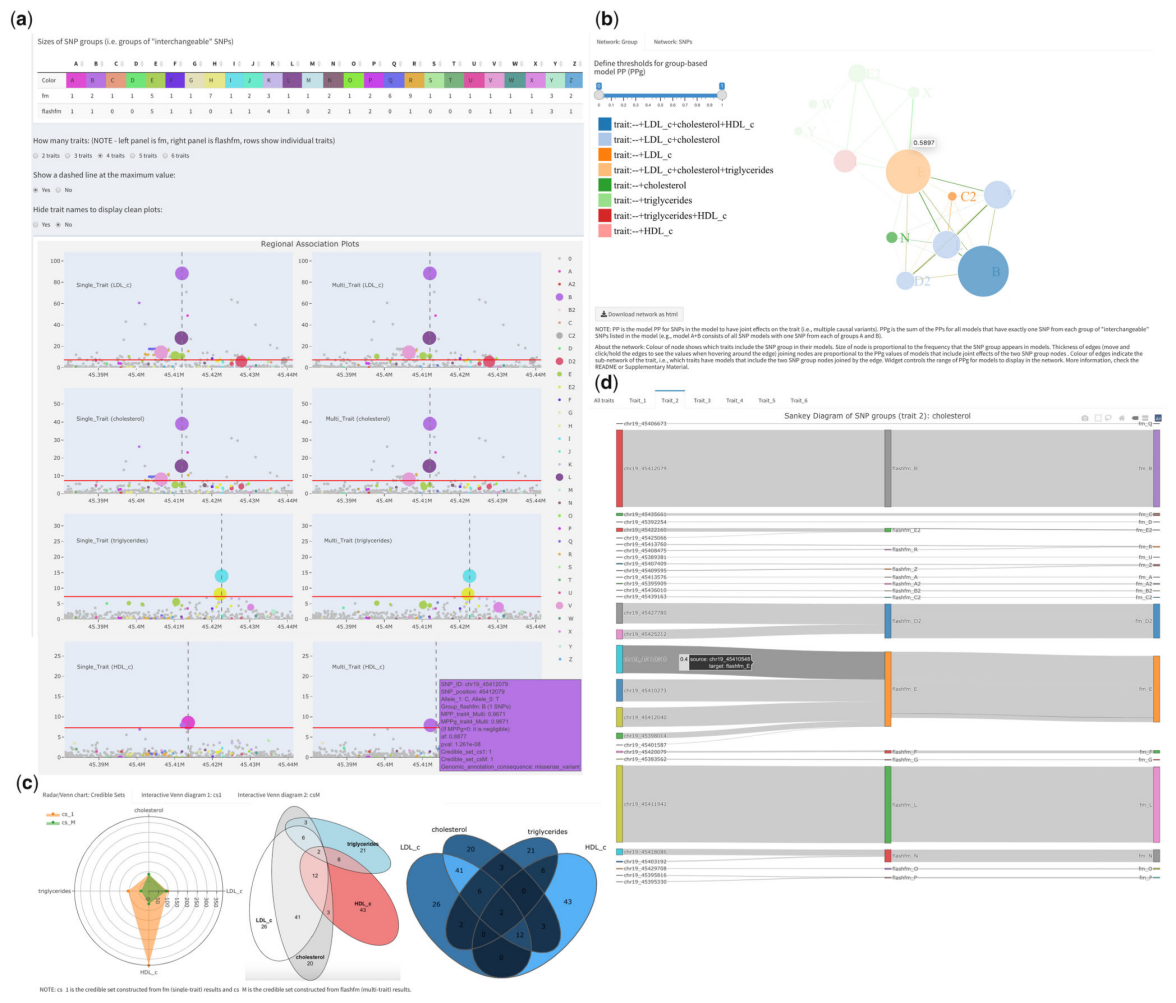
Selected *flashfm-ivis* interactive plots for four lipid traits from Ugandan GWAS data (Gurdasani *et al.,* 2019) in the APOE region (Hernandez *et al*., 2021). (**A**) Interactive and linked regional association plots with integrated fine-mapping results. Four lipid traits [LDL-cholesterol, total cholesterol (TC), triglycerides (TG) and HDL-cholesterol] were single- (left panel) and multi-trait (right panel) fine-mapped; the rows correspond to each trait: LDL, TC, TG and HDL. The regional association plots (–log10(*p*) against variant position) integrate the variant MPPs (see Section 2.2) from fine-mapping, by displaying the diameter of each variant point as proportional to its MPP. Colours indicate variant groups, which consist of variants in high LD and could be treated as interchangeable in the causal models. Users may click on particular groups to focus on (or to remove) in all plots or they may draw their selection of points in one plot as a focus for all plots. Hovering over a variant gives information such as allele frequency, most severe consequence [VEP, version 85 (McLaren *et al*., 2016; http://www.nealelab.is/uk-biobank)] and fine-mapping PP results. Genes within the region are also displayed. (**B**) Network diagram of variant group causal models. Variant group models are displayed for the four lipid traits. Each node represents a variant group and edges between nodes indicate variant groups that appear together in a model; only models with PPg (as defined in Section 2.4) within the range of the widget selection are included. The colours of the nodes indicate which traits include the variant group in their models, and their diameter is proportional to the frequency that they appear in models over all traits. Edge colours indicate which traits include the pair of variant groups in a model, while edge thickness is proportional to the PPg values. (**C**) Radar Chart and Venn diagrams of credible sets for traits. These plots show and compare the number of variants that appear in multiple credible sets for different methods and traits. For example, in the Venn diagram, the four traits share two variants in their credible sets constructed from flashfm (easily seen in area-proportional Venn diagram), while the Radar chart compares the total number of variants based on FINEMAP (cs_1) and flashfm (cs_M). Hovering on a segment in the interactive Venn diagram shows the variant ids and details of each segment are in a downloadable table. (**D**) Sankey diagram connects individual variant ids and their groups in flashfm or FINEMAP (fm). This links the individual variants and their groups in different methods. The size of the edge reflects the MPP value of the variant in each trait (e.g. trait_2 cholesterol here, users can interact with the plots to show the values)

Key features of *flashfm-ivis* are shown in Figure 1 (also listed in Supplementary Table S2), with an overview here:

- Interactive visualizations encourage users to compare results for multiple traits.
- Downloadable summaries from different plots, based on users’ selections (e.g. credible sets, variant groups, etc.), simplify complex results and give insight for further analyses (e.g. discovering shared variants of credible sets for different trait combinations).
- Flexible ways of viewing the joint effects of variants on one or more traits.

## 2 Implementation

*Flashfm-ivis* is currently built in R and minimizes the complex (inter-)dependency with other R packages (Supplementary Table S2), making it a standalone tool and therefore easier to maintain in the long-term. The web-based version (http://shiny.mrc-bsu.cam.ac.uk/apps/flashfm-ivis/) does not require users to have any programming skills, encouraging a wide spectrum of researchers to interact with and visualize their own results. It includes six Dashboard tabs that give access to various comparisons and summaries, links to download all codes (https://github.com/fz-cambridge/flashfm-ivis) and a link to *finemap-ivis* (http://shiny.mrc-bsu.cam.ac.uk/apps/finemap-ivis/).

### 2.1 Data inputs

To illustrate its main features, flashfm-ivis includes a pre-loaded example from a cardiometabolic GWAS of a Ugandan cohort (Gurdasani *et al*., 2019) for four lipid traits and the *APOE* region (Hernandez *et al*., 2021; Supplementary Fig. S1).

Users may view both single and multi-trait fine-mapping results, by uploading their output from flashfm and its input files to *flashfm-ivis* (details in Supplementary Material). To view only single-trait fine-mapping results from FINEMAP (Supplementary Fig. S13), users may upload the standard FINEMAP output files to *finemap-ivis*.

A sub-dashboard (Supplementary Figs S1 and S12) is available for users to verify their input. All plots are interactive, allowing the user to control the regions displayed in regional association plots and to drag network nodes to change the perspective of plots. A genes panel shows the positions of the genes (Build 37, NCBI Reference Sequence database; O’Leary *et al*., 2016) within the region. An overview of key plots follows (also see Supplementary Table S2, all detailed plots in Supplementary Material).

### 2.2 Control widgets on the sidebar panel

Control widgets allow users to refine their data selection and then focus on key results; both credible sets and *MPP* (marginal PP of variant causality—the PP that the variant is included in any model) thresholds can be controlled (Supplementary Fig. S2).

### 2.3 Fine-map integrated regional association plots

Individual regional association plots are presented for each trait and MPPs from fine-mapping are shown by the dot size (Supplementary Fig. S2). In Supplementary Figure S8, all regional association plots are linked together for easy comparison between traits and between methods—users may select a subset of variants to focus on in all plots.

### 2.4 Network plots of joint variant effects

Supplementary Figures S4–S7 present both variant group- and individual-variant dynamic networks. The key features are: (1) users interact with the networks by defining the PP thresholds for variant models, so that the network can be expanded or refined; (2) the sizes and colours of both nodes and edges indicate evidence strength and associated traits, as detailed in Supplementary Table S2 and (3) sub-networks can be formed and moved. When viewing models based on variant groups, we consider the group model PP (PPg), which is the sum of the PPs for all models that have exactly one variant from each group listed in the model; for example, model A + B consists of all variant models with one variant from each of groups A and B (i.e. fm in ‘Ch_1_Single_trait’ and flashfm in ‘Ch_2_Multi_trait’).

### 2.5 Spider/radar diagram of credible set sizes

In Figure 1 and Supplementary Figure S9, the spider chart compares the number of variants in credible sets of fm/flashfm methods based on different traits.

### 2.6 Venn diagrams of shared potential causal variants

We provide area-proportional Venn diagrams that give an intuitive view of the degree of overlap between credible sets of the traits (Fig. 1 and Supplementary Fig. S9). Interactive Venn diagrams are also available, as well as allow users to view and download lists of variants in each combination of overlapping credible sets (Supplementary Fig. S10).

### 2.7 Sankey diagrams of variant groups

In Figure 1 and Supplementary Figure S11, Sankey flowcharts show the connections between variants and variant groups in models from the fm/flashfm methods and based on different traits.

## 3 Conclusion

We provide a user-friendly fully interactive web tool, *flashfm-ivis,* that is accessible without any programming knowledge; it is available as an R package for those who prefer to run it from their own machine. It promotes exploration of fine-mapping results of several traits and helps with interpretation, as well as identification of variants for further follow-up. Users can interact with plots and decide on the final version of publication-ready plots for download. *Flashfm-ivis* output, such as lists of variants in credible sets for selected traits, is easily downloaded for further follow-up. If users are interested in only a single trait, there is an option to produce plots based on FINEMAP output only, *finemap-ivis*. We hope that *flashfm-ivis* will become standard practice for exploring fine-mapping results and will contribute to revealing the underlying mechanisms of diseases.

## Supplementary Material

Supplementary File

## Figures and Tables

**Table 1:** Comparison of visualization features and tools for GWAS and fine-mapping results

Features and tools	Flashfm-ivis	echolocatoR	PAINTOR-CANVIS	PheGWAS	LcusZoom.js	Cgmisc	LDlink	Assocplots	IntAssoPlot
Input data in/from different formats/methods	+								
Display fine-mapping results	+	+	+						
Multi-panel comparison with linked data	+	+						+	
Joint SNP effects (fine-mapping model PPs)	+								
Interactive features/tools	+			+	+		+	+	
Download outputs/plots	+	+	+	+			+		
A standalone web or R package	+			+	+		+		
Regional association plot of GWAS results	+	+	+	+	+	+	+	+	+
Scatter plot of SNP PPs (MPP) only	+^a^	+	+						
Regional association plot with SNP PPs	+								
LD with lead SNP	+	+			+	+	+^b^		+
LD heatmap and plot	+		+				+^b^		+
Link between GWAS and LD matrix	+								+
Integrated display of multiple GWAS	+			+				+	+
No programming knowledge required	+				+		+		
Allow iPad or touch screen	+								
Can be used with other R/Python packages	+	+		+	+	+			
Reference	+	Schilder *et al*., 2022	Kichaev *et al*., 2017	George *et al*., 2020	Boughton *et al*., 2021; Pruim *et al*., 2010	Kierczak *et al*., 2015	Machiela and Chanock, 2015	Khramtsova and Stranger, 2017	He *et al*., 2020

*Note:* A ‘+’ indicates that the tool has the feature, possibly with a few modifications.

a *Flashfm-ivis* integrates the SNP PPs with the regional association plots.

b LDlink only uses built-in reference panels.

## Data Availability

Data results that appear in our examples are available from the public on-line Google Drive link provided at https://github.com/fz-cambridge/flashfm-ivis.

## References

[R1] Benner C (2016). FINEMAP: efficient variable selection using summary data from genome-wide association studies. Bioinformatics.

[R2] Boughton AP (2021). LocusZoom.js: interactive and embeddable visualization of genetic association study results. Bioinformatics.

[R3] Claussnitzer M (2020). A brief history of human disease genetics. Nature.

[R4] George G (2020). PheGWAS: a new dimension to visualize GWAS across multiple phenotypes. Bioinformatics.

[R5] Gurdasani D (2019). Uganda genome resource enables insights into population history and genomic discovery in Africa. Cell.

[R6] He F (2020). IntAssoPlot: an R package for integrated visualization of genome-wide association study results with gene structure and linkage disequilibrium matrix. Front Genet.

[R7] Hernandez N (2021). The flashfm approach for fine-mapping multiple quantitative traits. Nat Commun.

[R8] Hutchinson A (2020). Fine-mapping genetic associations. Hum Mol Genet.

[R9] Khramtsova EA, Stranger BE (2017). Assocplots: a python package for static and interactive visualization of multiple-group GWAS results. Bioinformatics.

[R10] Kichaev G (2017). Improved methods for multi-trait fine mapping of pleiotropic risk loci. Bioinformatics.

[R11] Kierczak M (2015). Cgmisc: enhanced genome-wide association analyses and visualization. Bioinformatics.

[R12] Machiela MJ, Chanock SJ (2015). LDlink: a web-based application for exploring population-specific haplotype structure and linking correlated alleles of possible functional variants. Bioinformatics.

[R13] McLaren W (2016). The ensembl variant effect predictor. Genome Biol.

[R14] Newcombe PJ (2016). JAM: a scalable Bayesian framework for joint analysis of marginal SNP effects. Genet Epidemiol.

[R15] O’Leary NA (2016). Reference sequence (RefSeq) database at NCBI: current status taxonomic expansion, and functional annotation. Nucleic Acids Res.

[R16] Pruim RJ (2010). LocusZoom: regional visualization of genome-wide association scan results. Bioinformatics.

[R17] Schilder BM (2022). echolocatoR: an automated end-to-end statistical and functional genomic fine-mapping pipeline. Bioinformatics.

[R18] Spain SL, Barrett JC (2015). Strategies for fine-mapping complex traits. Hum Mol Genet.

