# Supplementary material for: Flashfm-ivis: interactive visualization for fine-mapping of multiple quantitative traits
Source: Bioinformatics. Author manuscript; Available in PMC 2022 Sep 8. (PMC9438951; doi:10.1093/bioinformatics/btac453)
Supplement: Supplementary File [file EMS151162-supplement-Supplementary_File.docx]

**Supplementary Material:**

***flashfm-ivis*: interactive visualisation for fine-mapping of multiple quantitative traits**

Feng Zhou, Adam S Butterworth and Jennifer L Asimit

**SUPPLEMENTARY TABLE_S1.** Other available GWAS visualisation software packages

| **Name of tools** | **Software link** | **Citation** | **Purpose and description** |
| --- | --- | --- | --- |
| Assocplots | https://github.com/khramts/assocplots | Khramtsova and Stranger, 2017,  *Bioinformatics* | A python package for static and interactive visualisation of multiple-group GWAS results |
| cgmisc | https://github.com/cgmisc-team/cgmisc | Kierczak et al., 2015,  *Bioinformatics* | An R package that enables enhanced data analysis and visualisation of results from GWAS |
| echolocatoR | https://github.com/RajLabMSSM/echolocatoR | Schilder et al., 2021,  *Bioinformatics* | Automated statistical and functional fine-mapping with extensive access to genome-wide datasets |
| FIVEx | https://fivex.sph.umich.edu  https://github.com/statgen/fivex/ | Kwong et al., 2021,  *Bioinformatics* | A web browser tool for visualising genotypes, expression and splice QTL (cis-eQTL and cis-sQTL) datasets |
| GAPIT | https://www.maizegenetics.net/gapit  https://github.com/jiabowang/GAPIT3 | Lipka et al., 2012, *Bioinformatics*  Wang and Zhang, 2021,  *Genomics, Proteomics and Bioinformatics (GPB)* | An R package that performs a GWAS and genome prediction (or selection) |
| GSDS | http://gsds.cbi.pku.edu.cn | Hu et al., 2015, *Bioinformatics* | Visualising genes' structure and annotated features helps biologists to investigate their function and evolution intuitively |
| Haploview | https://www.broadinstitute.org/haploview/haploview | Barrett et al., 2005, *Bioinformatics* | Haploview is designed to simplify and speed up haplotype analysis by providing a common interface to several tasks relating to such analyses |
| IntAssoPlot | https://github.com/whweve/IntAssoPlot | He et al., 2020, *frontiers in Genetics* | Visualise GWAS with Gene Annotation and Linkage Disequilibrium |
| JBrowse | https://jbrowse.org/jb2/ | Buels et al., 2016, *Genome Biology* | A fast and full-featured genome browser built with JavaScript and HTML5 |
| LDlink | https://ldlink.nci.nih.gov/?tab=home  https://github.com/CBIIT/nci-webtools-dceg-linkage | Machiela and Chanock, 2015,  *Bioinformatics* | A suite of web-based applications designed to interrogate LD easily and efficiently in population groups |
| LocusZoom | http://locuszoom.org | Pruim et al., 2010,  *Bioinformatics* | A suite of tools to provide fast visualisation of GWAS results for research and publication |
| LocusZoom.js | https://github.com/statgen/locuszoom/releases/tag/v0.12.0 | Boughton et al., 2021,  *Bioinformatics* | A JavaScript implementation of LocusZoom to make LocusZoom embeddable and interactive |
| PAINTOR-CANVIS | https://github.com/gkichaev/PAINTOR_V3.0/tree/master/CANVIS | Kichaev et al., 2017, *Bioinformatics* | A visualisation tool utilized for the output of PAINTOR |
| PheGWAS | https://github.com/georgeg0/PheGWAS | George et al., 2020, *Bioinformatics* | Three-dimensional approach to dynamically visualise GWAS across multiple phenotypes |
| ShinyGPA | https://github.com/dongjunchung/GPA | Kortemeier et al., 2018, *PLoS One* | An interactive visualisation toolkit for investigating pleiotropic architecture using GWAS datasets |
| Zbrowse | https://github.com/baxterlabZbrowse/ZBrowse | Ziegler et al., 2015,  *PeerJ Comput. Sci.* | An interactive GWAS viewer focused on comparing results across traits and other variables |

**SUPPLEMENTARY TABLE_S2.**Summary of key functions implemented in *flashfm-ivis*

| **Dashboard** | **Charts** | **Functions** | **Features** |
| --- | --- | --- | --- |
| GWAS summary statistics (Ch_0 Input Values) | Interactive regional association plots | + Colour: SNP Group  + Size: MPP value  + X axis: SNP location  + Y axis: P-value  + Widget_1: credible sets selection  + Widget_2: define MPP values  + Legend: user selection of SNP Group | Interactive tools:  + Download plots directly  + Box or Lasso Selection  + Zoom In and Out  + Show and compare data on hover  + Data selection via legend element  + Links to extra widgets |
| **See Figures S2 and S3** |  |  |  |
|  |  |  |  |
|  |  |  |  |
|  |  |  |  |
|  |  |  |  |
|  | Genes | + Show positions of genes within the region  + Widget: Refine the region to zoom in on genes in a portion of the region | Flexible ways to view genes in the region of the regional association plots |
|  | Coloured SNP Group | + Colour: link to the SNP Group in plots  + Value: number of SNPs in the group | Possible implementation: user can download the table directly |
|  |  |  |  |
|  | Interactive and selective LD plot | + Heatmap: an initial overview of LD  + LD plot: show selected SNPs | Interactive and dynamic features |
|  |  |  |  |
| Single trait fine mapping network (Ch_1 Single trait) | Group-based network | + Widget: define PP values  + Colour of node: traits that include the SNP/group in their models  + Size of node: frequency that the SNP/group appears in models  + Thickness of edge: PP values  + Colour of edge: sub-network of trait | Interactive tools:  + Define PP values  + Understanding sub-networks based on colours, nodes, and links |
| **See Figures S4 and S5** | Individual SNP-based network |  |  |
| Multi trait fine mapping network (Ch_2 Multi trait) | Group-based network |  |  |
| **See Figures S6 and S7** | Individual SNP-based network |  |  |
| Linked data comparison plots (Ch_3 Comparison (Linked)) | Coloured SNP Group | + Compare linked regional association plots across all traits  + All colours of SNP Groups are consistent in different plots  + Users’ own selections of data | Combined all interactive tools as above, but also linking selected data of different plots together |
| **See Figure S8** | Coloured and linked regional association plots |  |  |
|  |  |  |  |
| Method comparison plots (Ch_4 Comparison (Methods)) | Radar chart of credible sets | + Comparison of credible sets from different methods  + Intuitive view of shared variants between credible sets  + Show individual SNPs in different joint sets | Allow users to interact with the diagrams and understand (as well as download) individual SNPs in each joint set |
| **See Figures S9 and S10** | Area-proportional Venn diagram of credible sets  Interactive Venn diagram of credible sets |  |  |
| Trait comparison plots (Ch_5 Comparison (Traits)) | All trait combined Sankey diagram | + Comparison of SNP groups in different traits  + Show detailed connections between different groups in two methods | Allow users to focus on the main SNPs in particular SNP Groups based on different methods |
| **See Figures S11** | Individual traits of Sankey diagram |  |  |
|  |  |  |  |

NOTE: This initial version of flashfm-ivis is built completely in R but can be extended to other platforms (such as Python) for a more diversified audience with different backgrounds. It is designed to minimise the complex (inter-)dependency with other R packages, making it a standalone tool, so it will be easier to maintain in the long-term. Some functions require a few existing commonly-used open-source packages in the R community (see CRAN.R-project.org): plotly (Sievert, 2020; https://plotly.com/r/), ggplot2 (Wickham, 2011; https://ggplot2.tidyverse.org), shiny (Verity, et al., 2017; https://shiny.rstudio.com and https://cran.r-project.org/package=shiny), shinydashboard (Chang, 2015; https://rstudio.github.io/shinydashboard/), DT (https://rstudio.github.io/DT/), gaston (https://cran.r-project.org/package=gaston), randomcoloR (https://cran.r-project.org/package=randomcoloR), networkD3 (Allaire, et al., 2017; https://cran.r-project.org/package=networkD3), ggVennDiagram (Gao, et al., 2021; https://cran.r-project.org/package=ggVennDiagram), dplyr (https://dplyr.tidyverse.org), igraph (Csardi and Nepusz, 2016; https://igraph.org/r/), stringr (https://cran.r-project.org/package=stringr).

**Flashfm-ivis (FLexible And Shared information Fine-Mapping-Interactive VISualisation)**

***Instructions: Input files***

The key variables and their names should be fixed as follows:

Finemap-ivis: <http://shiny.mrc-bsu.cam.ac.uk/apps/finemap-ivis/>

Users can upload the four standard input/output files from [FINEMAP](http://www.christianbenner.com/) ([Benner et al. 2016](https://academic.oup.com/bioinformatics/article/32/10/1493/1743040)). They can use any names for the files, but the file extensions should be fixed as follows:

1. Anyname.config
2. Anyname.ld
3. Anyname.snp
4. Anyname.z

Flashfm-ivis: <http://shiny.mrc-bsu.cam.ac.uk/apps/flashfm-ivis/>

This requires output from running multi-trait fine-mapping with [flashfm](https://jennasimit.github.io/flashfm/) ([Hernandez et al. 2021](https://www.nature.com/articles/s41467-021-26364-y)).

Depending on the following three situations (i.e. options on the webpage sidebar), users can upload different data files by using the “Browse” button:

1. **Single RData file:** if users save all variables (i.e. items 1.a – 1.d) below in a single RData file, they can upload this file directly to the server, but please make sure the list and variable names are fixed as follows:
   1. A list named GWAS, where GWAS[[m]] is the data.frame for the m^th^ trait. Inside each data.frame GWAS[[m]], the variables (columns) must be given in the following order:
      1. $rs: name or rsid of SNP
      2. $chr: chromosome number (to mark the x-axis label in the plots)
      3. $ps: base-pair position of SNP
      4. $allele1: allele1 of SNP (effect allele)
      5. $allele0: allele0 or allele2 of SNP (non-effect allele)
      6. $beta: single-SNP effect estimate
      7. $se: standard error of beta
      8. $pval: p-value
      9. $af: effect allele frequency or minor allele frequency (MAF)
      10. $no: sample size at each SNP (i.e. if unavailable, assign zero or NA to this column)
   2. A matrix (i.e. not a list) of LD with column and row names specified by the SNPs. The SNP names must match those in GWAS[[m]]$rs, as above.
   3. A list of standard flashfm output mpp.pp, which contains the 4 usual sub-lists as:
      1. mpp.pp$MPP
      2. mpp.pp$MPPg
      3. mpp.pp$PP
      4. mpp.pp$PPg
   4. A list of standard flashfm output snpGroups, which contains the 3 usual sub-lists as:
      1. snpGroups$groups.fm
      2. snpGroups$groups.flashfm
      3. snpGroups$group.sizes
   5. PLEASE NOTE: users can use save(GWAS, LD, mpp.pp, snpGroups, file="filename.RData") to keep/store the above four datasets together from their R/RStudio workspace to one single .RData file and save it in their local working directory, then upload it to the webpage server.
   6. A demonstration YouTube video has been added to the web-based version README tab.
2. **FINEMAP with flashfm:** if users have separate files from different sources (e.g. FINEMAP and flashfm packages), then the files with different extensions must have the following variables with the fixed names:
   1. For each trait, a standard .z file from [FINEMAP](http://www.christianbenner.com/) for all individual traits, (please do not change the variable names, but the order of columns can be changed), which contains:
      1. $rsid
      2. $chromosome
      3. $position
      4. $allele1
      5. $allele2
      6. $maf
      7. $beta
      8. $se
   2. One .ld file that contains the standard LD matrix from FINEMAP (please do not change anything).
   3. One .RData file that was created and saved from the output of the flashfm R package, which contains the mpp.pp and snpGroups objects. (Please save both datasets directly in the .RData file by using save(mpp.pp, snpGroups, file="filename.RData"), do not combine them as a list).
   4. PLEASE NOTE: if users upload multiple .z files at the same time, please order the files from trait_1 to trait_m (regardless of the names of these .z files), since R-Shiny will read the first .z file sent to the server as trait 1, the second .z file as trait 2 and so on. (See the YouTube video for a detailed illustration.)
   5. A demonstration YouTube video has been added to the web-based version README tab.
3. **FLASHFMwithJAM:** similar to **FINEMAP with flashfm,** but uses the expanded JAM method in the [flashfm](https://jennasimit.github.io/flashfm/) R package. Save the mpp.pp and snpGroups objects to an .RData file, as described above. A similar YouTube video has been added to the web-based version.


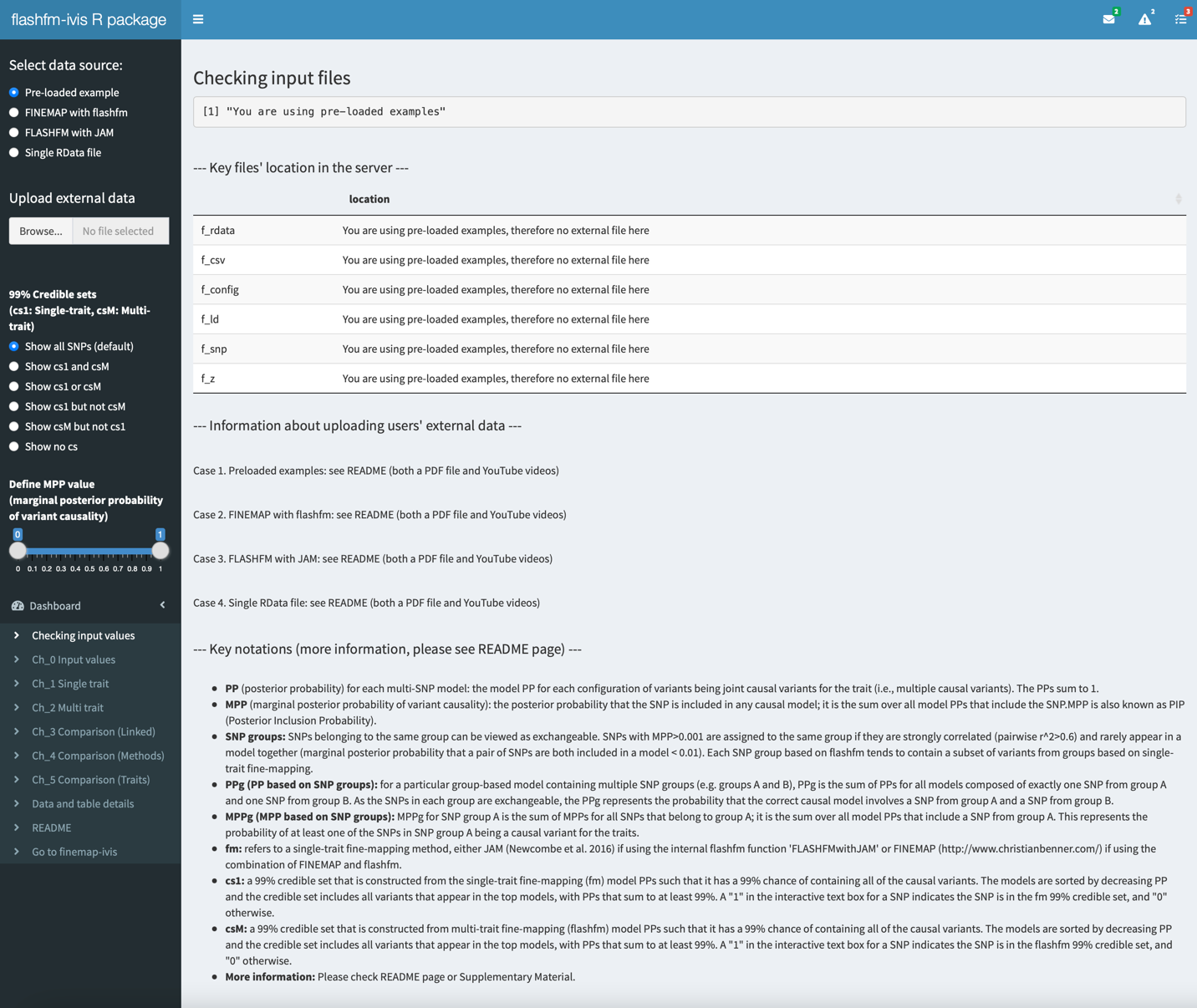


*Figure_S1: Data input page*

***Instructions: Available plots and data summaries***

All plots that are generated could be downloaded directly in a publication-ready format (the default is a PNG file of size 700 x 450 pixels). The file specifics may be controlled via the toImageButtonOptions configuration key. Possible formats: 'format': 'svg', # one of png, svg, jpeg, etc. More information: <https://plotly.com/python/configuration-options/#customizing-modebar-download-plot-button>.

The user may also interact with the plots to select different perspectives and download their selected version.

Below we refer to:

- **PP** (posterior probability) for each multi-SNP model: the model PP for each configuration of variants being joint causal variants for the trait (i.e., multiple causal variants). The PPs sum to 1.
- **MPP** (marginal posterior probability of variant causality): the posterior probability that the SNP is included in any causal model; it is the sum over all model PPs that include the SNP. MPP is also known as PIP (Posterior Inclusion Probability).
- **SNP groups:** SNPs belonging to the same group can be viewed as exchangeable. SNPs with MPP>0.001 are assigned to the same group if they are strongly correlated (pairwise r^2^>0.6) and rarely appear in a model together (marginal posterior probability that a pair of SNPs are both included in a model < 0.01). Each SNP group based on flashfm tends to contain a subset of variants from groups based on single-trait fine-mapping.
- **PPg (PP based on SNP groups):** for a particular group-based model containing multiple SNP groups (e.g. groups A and B), PPg is the sum of PPs for all models composed of exactly one SNP from group A and one SNP from group B. As the SNPs in each group are exchangeable, the PPg represents the probability that the correct causal model involves a SNP from group A and a SNP from group B.
- **MPPg (MPP based on SNP groups):** MPPg for SNP group A is the sum of MPPs for all SNPs that belong to group A; it is the sum over all model PPs that include a SNP from group A. This represents the probability of at least one of the SNPs in SNP group A being a causal variant for the traits.
- **fm** refers to a single-trait fine-mapping method, either JAM ([Newcombe et al. 2016](https://onlinelibrary.wiley.com/doi/full/10.1002/gepi.21953)) if using the internal [flashfm](https://jennasimit.github.io/flashfm/) function ‘FLASHFMwithJAM’ or [FINEMAP](http://www.christianbenner.com/) (<http://www.christianbenner.com/>) if using the combination of FINEMAP and flashfm.
- **cs1:** a 99% credible set that is constructed from the single-trait fine-mapping (fm) model PPs such that it has a 99% chance of containing all the causal variants. The models are sorted by decreasing PP and the credible set includes all variants that appear in the top models, with PPs that sum to at least 99%. A “1” in the interactive text box for a SNP indicates the SNP is in the fm 99% credible set, and “0” otherwise.
- **csM:** a 99% credible set that is constructed from multi-trait fine-mapping (flashfm) model PPs such that it has a 99% chance of containing all the causal variants. The models are sorted by decreasing PP and the credible set includes all variants that appear in the top models, with PPs that sum to at least 99%. A “1” in the interactive text box for a SNP indicates the SNP is in the flashfm 99% credible set, and “0” otherwise.

**Modebar icons that offer users different interactive features in dashboard plots**

| **Icon** | **Use** |
| --- | --- |
| **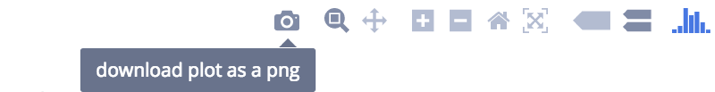** | Click on the camera icon and get/download the plot in PNG format. |
| **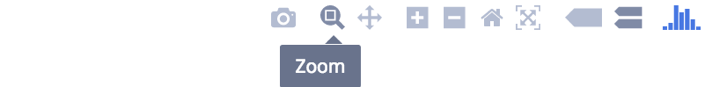** | Clicking this icon selects the Zoom mode. To zoom in on a region of a graph, click and hold your mouse, moving across the region. Release your mouse. To return to the original view, double-click anywhere on the plot. |
| **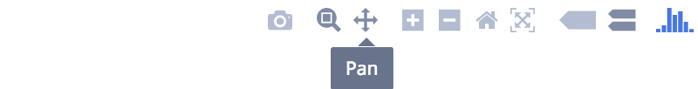** | To pan across regions of your graph, select the Pan mode. Click and hold your mouse to explore the data. Double-click anywhere to return to the original view. |
| **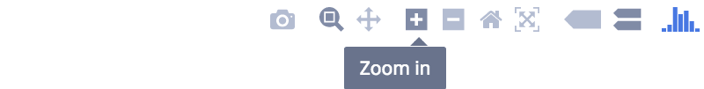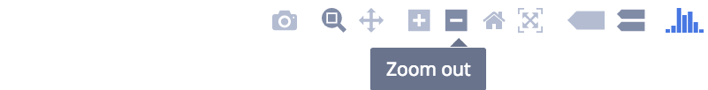** | You can zoom in and out by clicking on the + and - buttons. The plot keeps axes labels and annotations the same size to preserve readability. |
| **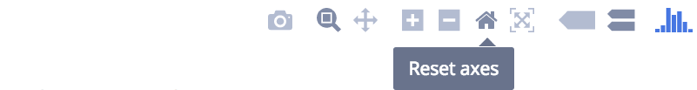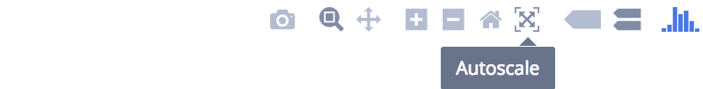** | Clicking this icon zooms to include your Axes Range, if this has been set. If it has not been set it zooms to a setting that is optimized to include all the viewable data, the same as if Autoscale had been clicked. |
| **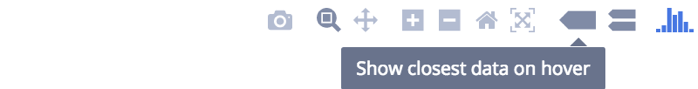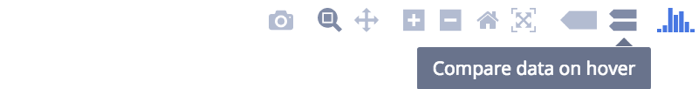** | One of these two buttons is always selected. Clicking ‘Show closest data on hover’ will display the data for just one point under the cursor. Clicking ‘Compare data on hover’ will show you the data for all points with the same x-value. |
| **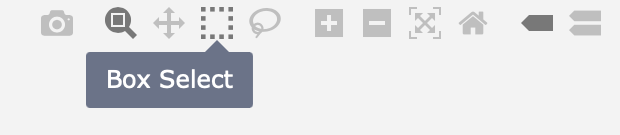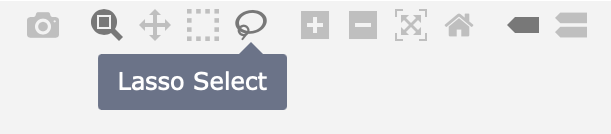** | These are basic selection tools. Box Select is to select data on the plot by creating a box, while the Lasso Select is used to draw with freehand selection. |
|  | **In general, by double-clicking anywhere on the plot, users can return to the original view.** |
| **More reference:** | <https://plotly.com/chart-studio-help/getting-to-know-the-plotly-modebar/> |

**Ch_0 Input Values – GWAS summary statistics and input values**

1. **Interactive regional association plots with integrated fine-mapping results – easily view SNPs based on both genetic association and probability of causality (from single-trait and multi-trait fine-mapping) in a familiar GWAS format**

The upper panel is for single-trait fine-mapping results and the lower panel is for multi-trait fine-mapping results. Within each panel, there is a tab for each trait. Within each panel tab a regional association plot (-log_10_(p) against SNP position) is displayed for each trait with the following additional features:

1. Hover over a point to see SNP details (SNP ID, alleles, allele frequency, etc. Please note, if MPPg is negligible, we show it as equal to 0.). There is also an option to view the most severe consequence (VEP^a,b^, version 85), by ticking a button; it is off by default due to the extra time that it takes to process and display this extra information. A “1” in the interactive text box for a SNP indicates the SNP is in the specified 99% credible set, and “0” otherwise.
2. Colour of points: SNP group membership according to fine-mapping results. SNPs belonging to the same group can be viewed as exchangeable. SNPs with MPP>0.001 are assigned to the same group if they have high LD (pairwise r^2^>0.6) and rarely appear in a model together (marginal posterior probability that a pair of SNPs are both included in a model < 0.01).
3. Size of points: proportional to fine-mapping posterior probabilities of SNP causality (referred to as MPP – Marginal Posterior Probability)
4. 99% credible set selection widget (see left sidebar ‘99% Credible sets’) – highlight SNPs based on inclusion/exclusion in credible sets from single-trait (i.e. defined as ‘cs1’ on the left sidebar) and/or multi-trait fine-mapping (i.e. defined as ‘csM’ on the left sidebar); can be combined with MPP widget.
5. MPP range widget (see left sidebar ‘Define MPP value’) – highlight SNPs that have MPP falling between the minimum and maximum sliders on the widget; can be combined with the credible set widget
6. Click on “Box Select” or “Lasso Select” to draw a box or a lasso (free drawing of any shape) around points to focus on and fade other points.
7. Click on “Zoom” (or Zoom In/out), then draw a box around points to zoom in and out for point selection.
8. Single click on a SNP group in the legend to remove all points belonging to that group.
9. Double click on a SNP group in the legend to remove all points not belonging to that group.
10. Click on “Pan” and then drag the plot to the left or right to change the centre of the plot.
11. Click on reset axis to res-set to the default plot
12. “Download plot as a PNG” option can download the plot to the local machine
13. “Autoscale” to adjust the view of the current plot

[a] McLaren et al. 2016;

[b] <http://www.nealelab.is/uk-biobank>

1. **Genes - positions of genes within the region that is in the regional association plots.**
2. At most 50 genes are displayed, aligned with the positions shown in the regional association plots; gene positions are based on build 37 from the NCBI Reference Sequence database (O’Leary et al. 2016.).
3. Zoom in on genes in a certain portion of the region by using the widget.
4. **Coloured SNP Groups – colours match those in the legend of the interactive regional association plots**
5. View SNP group sizes from both single and multi-trait fine-mapping
6. The table is adjusted automatically depending on the computer screen size, but users can also use the mouse/touchpad to scroll left or right of the table.
7. **Interactive and selective LD plot – heat map style plot of r^2^ between SNPs**
8. User selects which SNPs to include in the LD plot based on the credible set and MPP widgets that control the interactive regional association plots (see c and d of the interactive regional association plots)
9. Click on Zoom to focus on an area of the plot.
10. Other interactive features (e.g. Pan, Autoscale, Download, etc) are the same as in the regional association plots.
11. **Traditional LD.plot if there are few selected SNPs (i.e. when users click “99% Credible sets” control widget to show cs1 and/or csM) and a histogram if there are many selected SNPs (since the LD.plot function takes a long time to run when there are many SNPs)**
    1. Users can download the plot by clicking the button
    2. The plot will be updated according to the control options in the sidebar

**
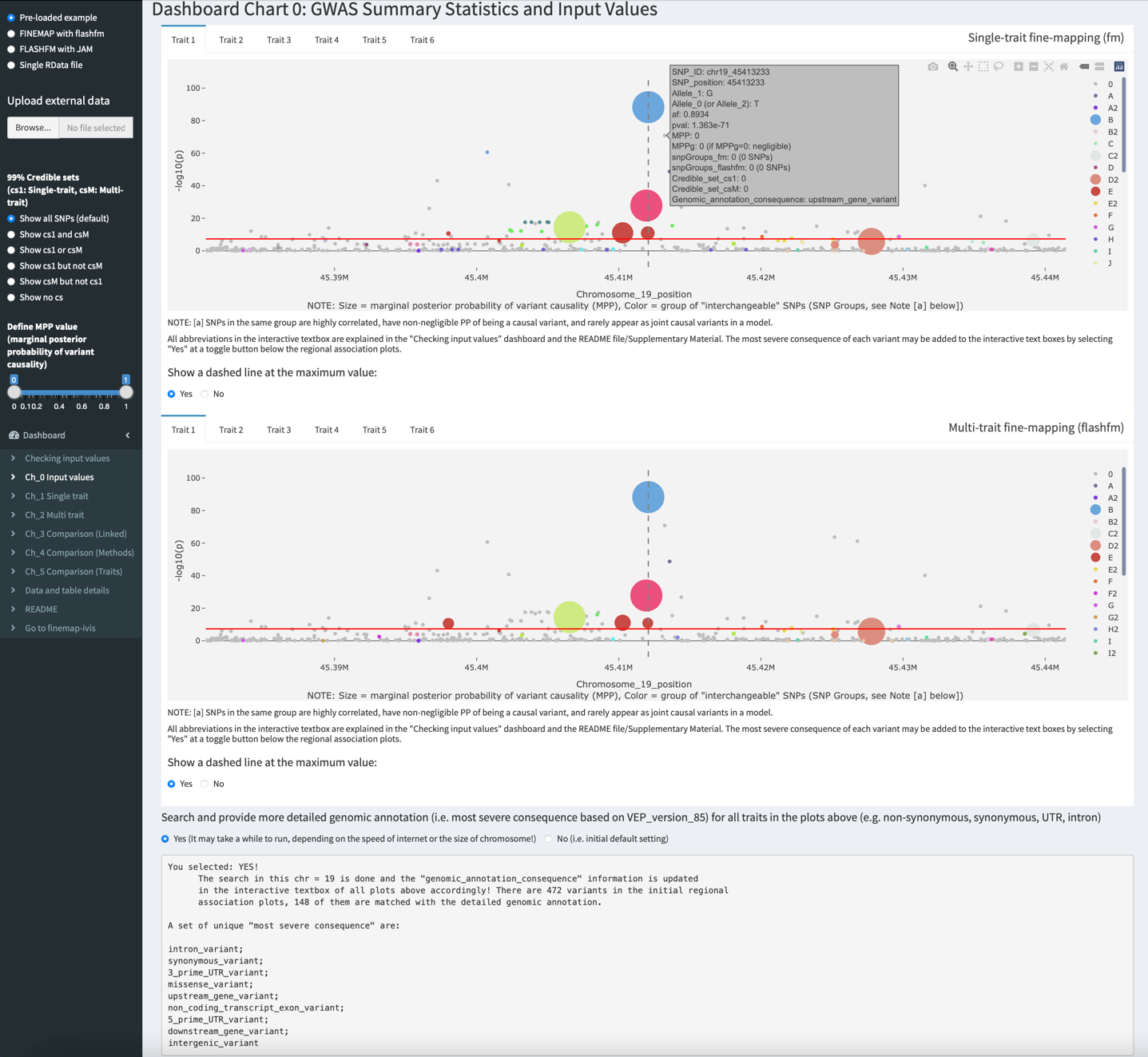
**

*Figure_S2: Ch_0 Input values (a)*

**
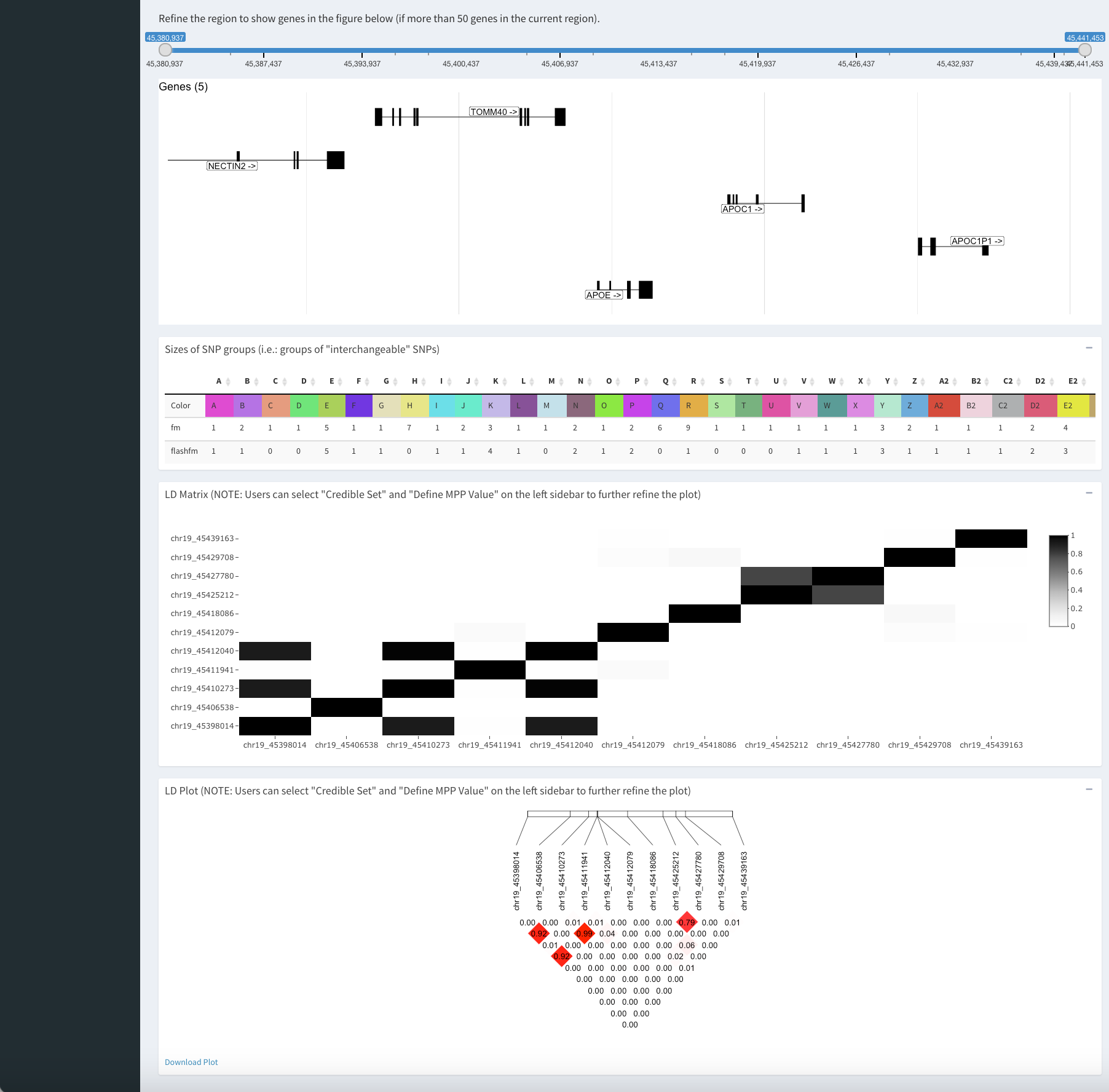
**

*Figure_S3: Ch_0 Input values (b). The LD Matrix and LD plot have been refined using the “99% credible set widget” set to “Show cs1 and csM” and the “Define MPP value” widget set to 0.02 to 1.*

**Ch_1 Single trait – Single-trait fine-mapping models**

**Group-based network – interactive visualisation of single-trait fine-mapping PP from models, to see which SNP groups tend to appear together in a model, having joint effects on a trait(s)**

1. Colour of node shows which traits include the SNP group in their models
2. Size of node is proportional to the frequency that the SNP group appears in models
3. Thickness of edges joining the nodes are proportional to the PPg values of models that include joint effects of the two SNP group nodes; hover over the edges to see the PPg values
4. Colour of edges indicate the sub-network of the trait, i.e., which traits have models that include the two SNP group nodes joined by the edge
5. Widget controls the range of PPg for models to display in the network
6. Nodes may be dragged to change the perspective of the plot
7. Users can scroll (Zoom in/out) the view of networks (i.e., to make the view larger or smaller to see the whole picture). Also, the view can be adjusted automatically depending on the size of the computer/tablet screen.
8. Users can download the network by clicking the button. Due to the interactive features of this network, the downloaded plot is a dynamic html format/webpage, but users can open this html file in their local machines and save/print the network as a static PDF file or use a screenshot to save it as a static PNG file.

**Individual SNP-based network – interactive visualisation of single-trait fine-mapping PP from models, to see which SNPs tend to appear together in a model, having joint effects on a trait(s)**

1. Same features as for the SNP group network
2. As there are many more SNP nodes than SNP group nodes, it is advised to have a higher minimum PP threshold to simplify and focus on the most likely models
3. There may be more than one sub-group network (e.g., depending on the PP value, two or more separate networks may be formed), therefore users can use their mouse or touchpad to scroll (Zoom in/out) of the view, in order to see all sub-networks.
4. Since it is a large network, SNPs that are not connected in a network (depending on the selected PP value) will be placed further from the centre, to focus on the main network.
5. Users can download the network by clicking the button. Due to the interactive feature of this network, the downloaded plot is a dynamic html format/webpage, but users can open this html file in their local machines and save/print the network as a static PDF file or use a screenshot to save it as a static PNG file.


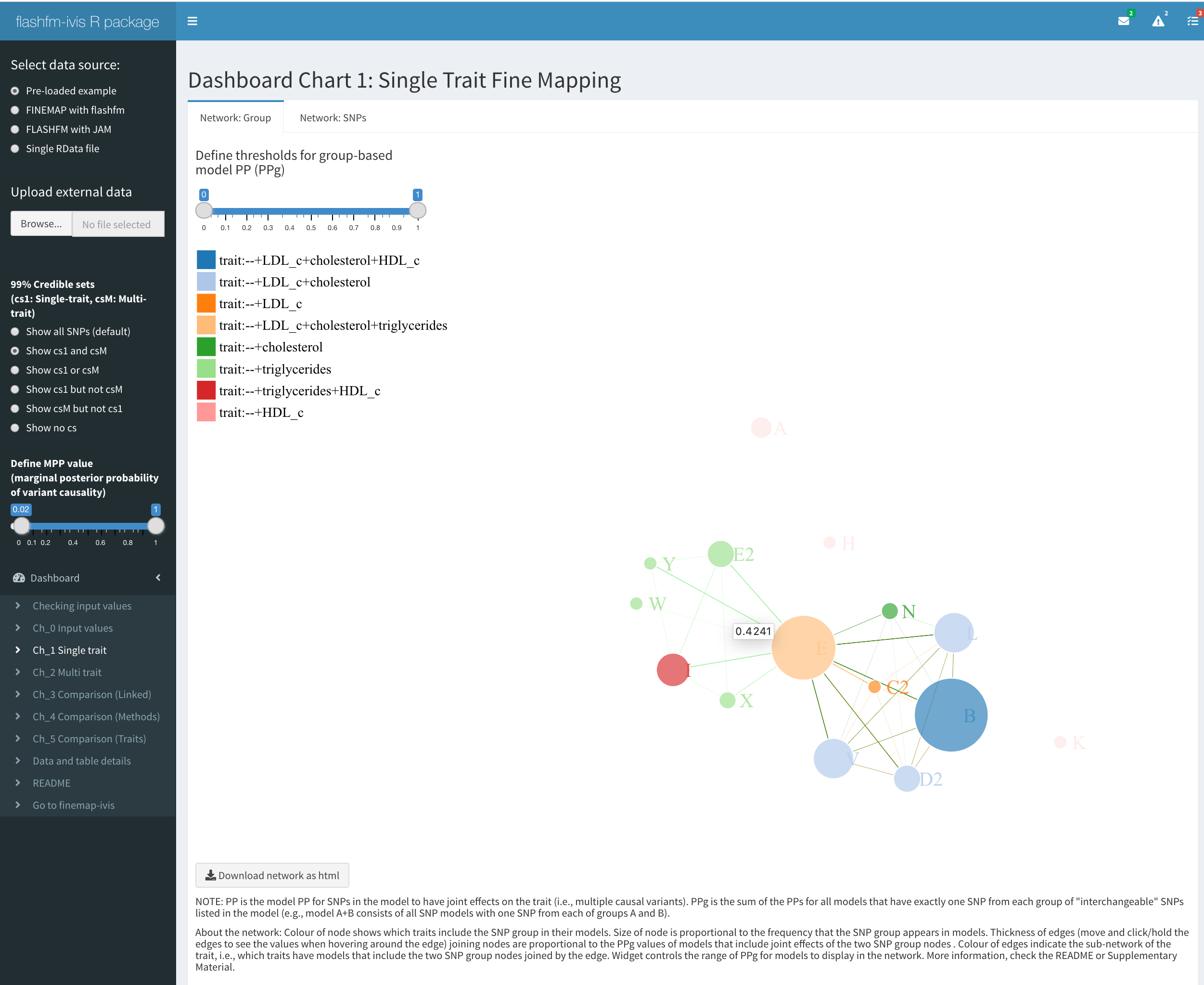


*Figure_S4: Ch_1_Single_trait (a)*

**
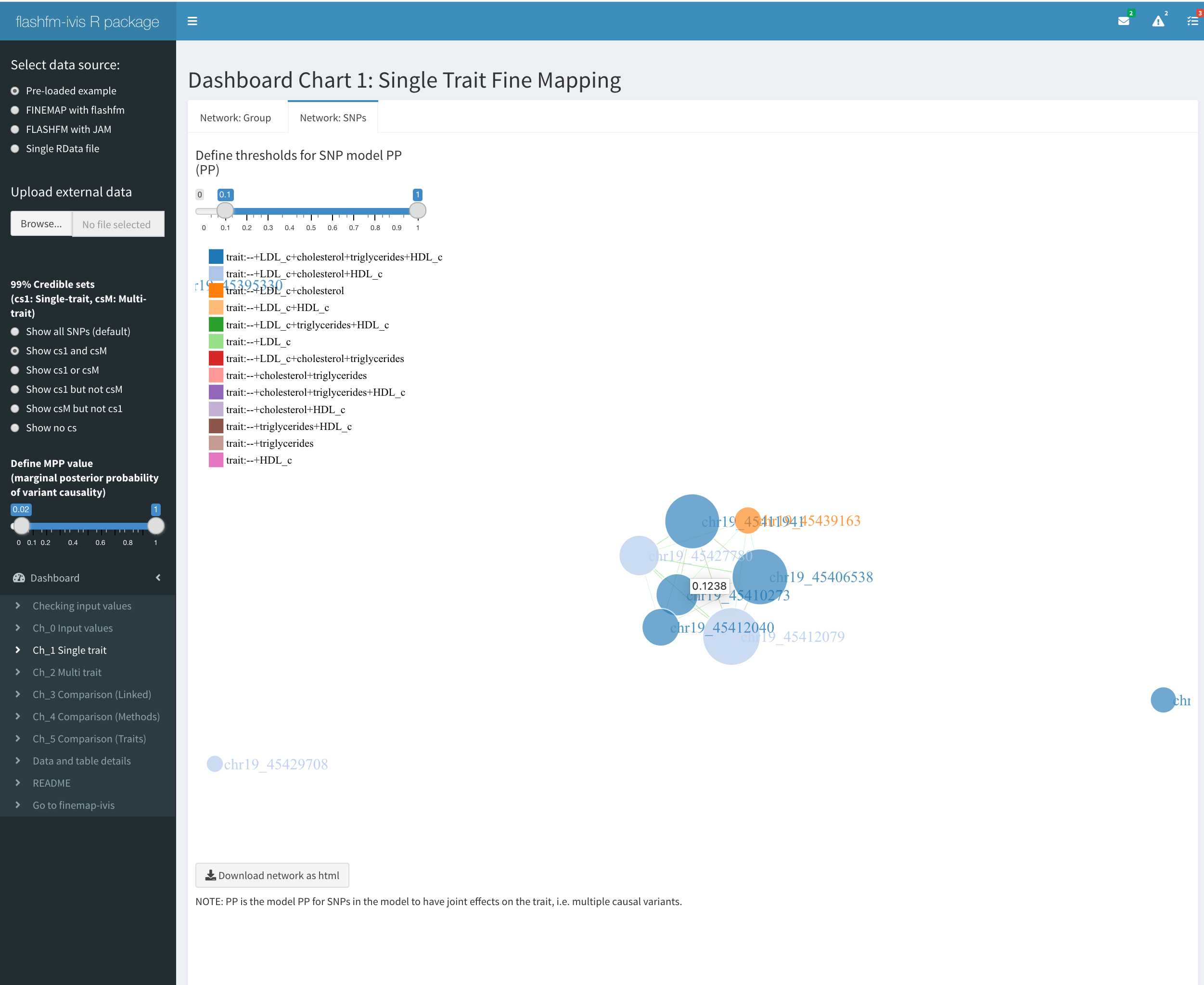
**

*Figure_S5: Ch_1_Single_trait (b)*

**Ch_2 Multi trait – Multi-trait fine-mapping models**

**Group-based network- interactive visualisation of multi-trait fine-mapping PP from models, to see which SNP groups tend to appear together in a model, having joint effects on a trait(s)**

1. Colour of node shows which traits include the SNP group in their models
2. Size of node is proportional to the frequency that the SNP group appears in models
3. Thickness of edges joining nodes are proportional to the PPg values; hover over the edges to see the PPg values
4. Colour of edges indicate the sub-network of the trait, i.e., which traits have models that include the two SNP group nodes joined by the edge
5. Widget controls the range of PPg for models to display in the network
6. Nodes may be dragged to change the perspective of the plot
7. Users can use their mouse or touchpad to scroll (Zoom in/out) the view of networks (i.e., to make the view larger or smaller to see the whole picture). Also, the view can be adjusted automatically depending on the size of the computer/tablet screen.
8. Users can download the network by clicking the button. Due to the interactive features of this network, the downloaded plot is a dynamic html format/webpage, but users can open this html file in their local machines and save/print the network as a static PDF file or use screenshot to save it as a static PNG file.

**Individual SNP-based network- interactive visualisation of multi-trait fine-mapping PP from models, to see which SNPs tend to appear together in a model, having joint effects on a trait(s)**

1. All the same features as for the SNP group network
2. As there are many more SNP nodes than SNP group nodes, it is advised to have a higher minimum PP to simplify and focus on the most likely models
3. There may be more than one sub-group network (e.g., depending on the PP value, two or more separate networks may be formed), therefore users can use their mouse or touchpad to scroll (Zoom in/out) of the view, in order to see all sub-networks.
4. Since it is a large network, SNPs will move far away from the centre if they are not connected in a network (depending on the selected PP value), in order to have a clear view of the main network.
5. Users can download the network by clicking the button. Due to the interactive features of this network, the downloaded plot is a dynamic html format/webpage, but users can open this html file in their local machines and save/print the network as a static PDF file or use a screenshot to save it as a static PNG file.


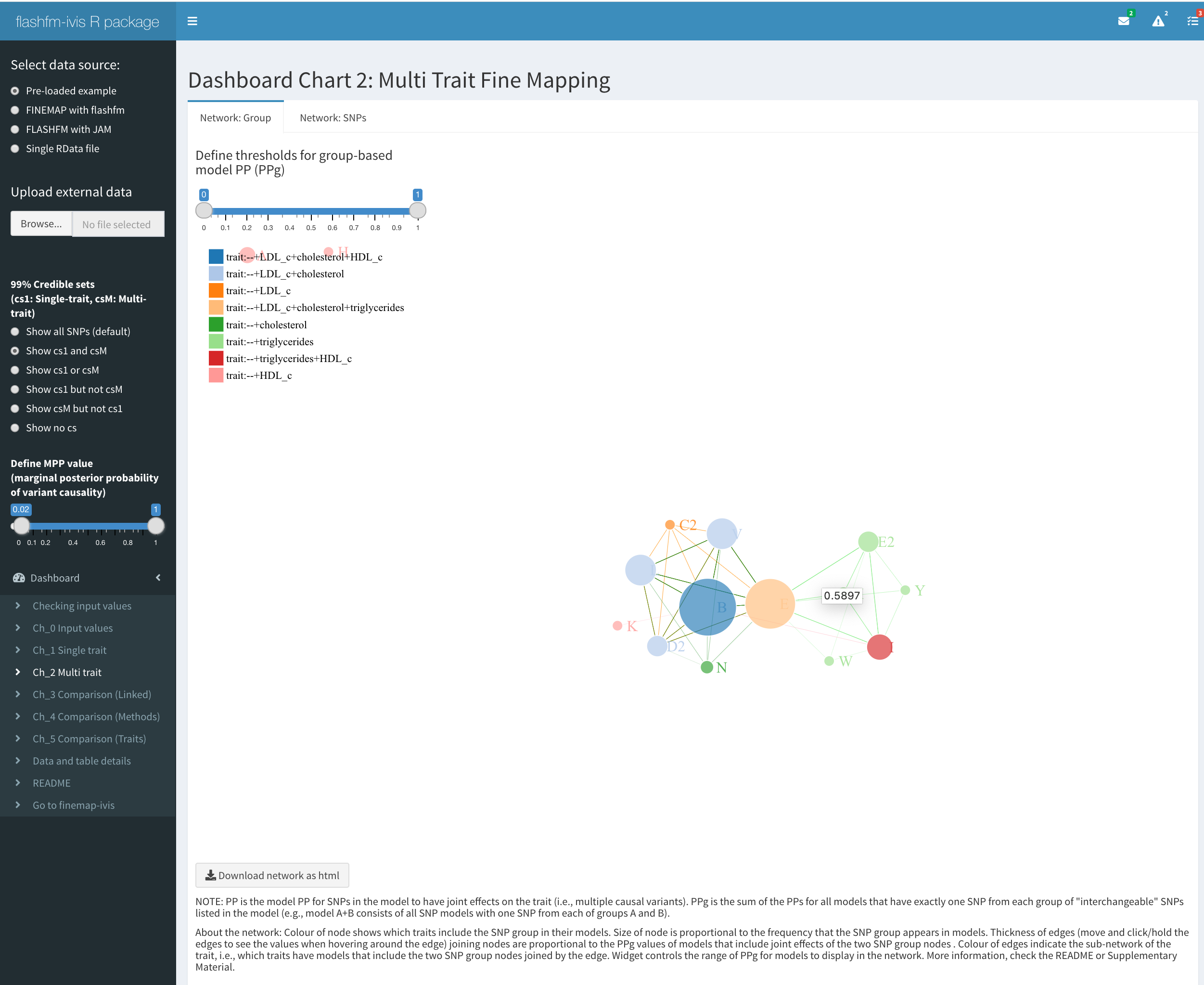


*Figure_S6: Ch_2_Multi_trait (a)*

**
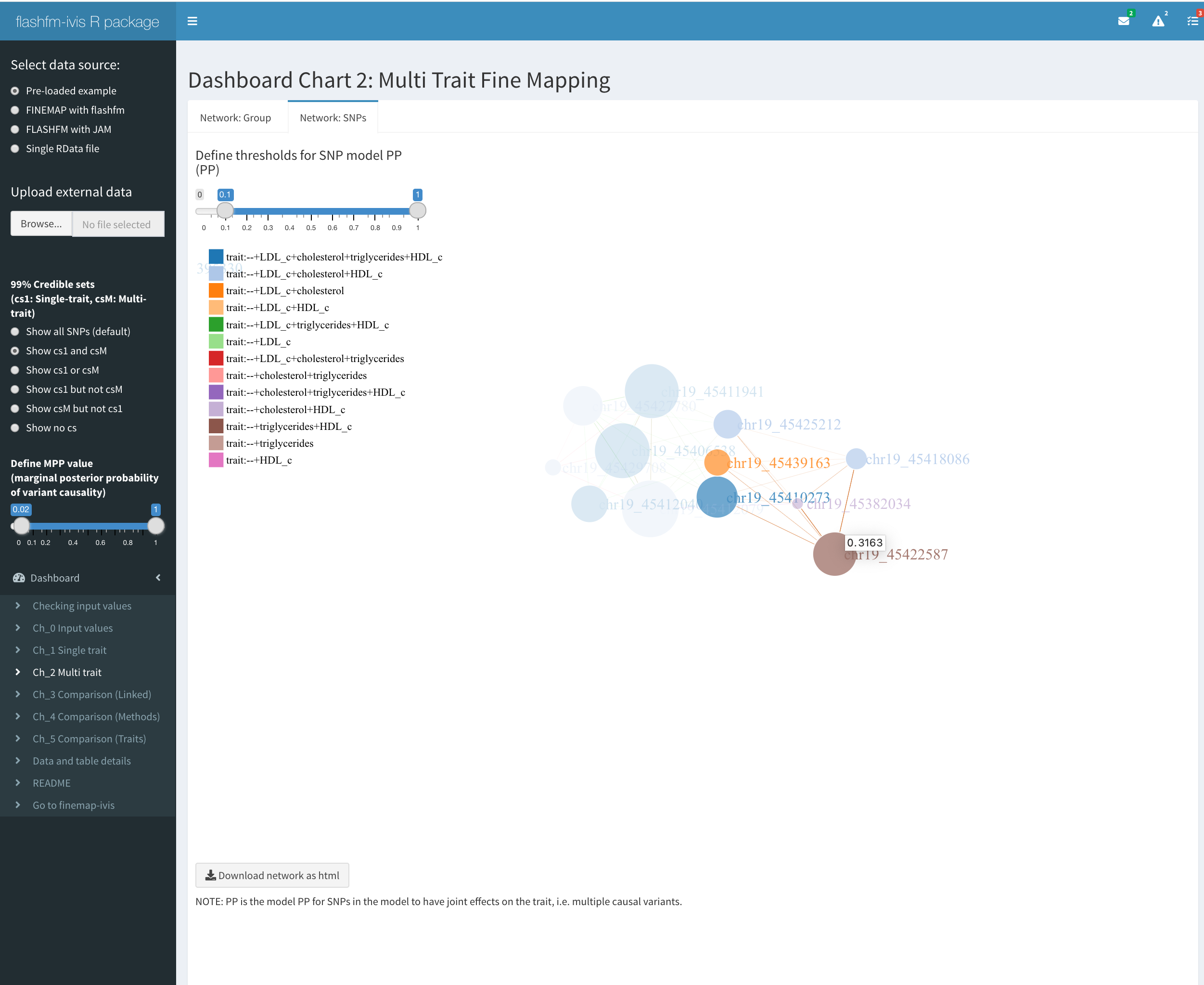
**

*Figure_S7: Ch_2_Multi_trait (b)*

**Ch_3 Comparison (Linked) – Multi-panel Integrated Fine-mapping regional association plots of traits for fm and flashfm**

**Coloured SNP Group– colours match those in the legend of the interactive regional association plots**

1. View SNP group sizes from both single and multi-trait fine-mapping
2. The table will be adjusted automatically depending on the computer screen size, but users can also use their mouse/touchpad to scroll left or right of the table.

**Coloured and linked regional association plots – view and interact with both GWAS and fine-mapping results**

The left panel is for single-trait fine-mapping results and the right panel is for multi-trait fine-mapping results. Each row shows individual trait results - a regional association plot (-log_10_(p) against SNP position) with the following additional features:

1. Hover over a point to see SNP details (SNP ID, alleles, allele frequency, etc. Please note, if MPPg is negligible, we show it as equal to 0.) or click on “Compare data on hover” to see details for several SNPs nearby at once. There is also an option to view the most severe consequence (VEP^a,b^, version 85), by ticking a button; it is off by default due to the extra time that it takes to process and display this extra information.
2. Colour of points: SNP group membership according to fine-mapping results. SNPs belonging to the same group can be viewed as exchangeable. SNPs with MPP>0.001 are assigned to the same group if they have high LD (pairwise r^2^>0.6) and rarely appear in a model together (marginal posterior probability that a pair of SNPs are both included in a model < 0.01).
3. Size of points: proportional to fine-mapping posterior probabilities of SNP causality (referred to as MPP – Marginal Posterior Probability)
4. Click on “Box Select” or “Lasso Select” to draw a box or a lasso (free drawing of any shape) around points to focus on and fade other points. Automatically, this same set of points will become the focus in all other plots allowing simplified comparisons.
5. Click on “Zoom”, then draw a box around points to zoom in and out for point selection.
6. Double click on a SNP group in the legend to remove all points not belonging to that group. Double click again to show all points not belonging to the SNP group
7. Click on “Pan” and then drag the plot to the left or right to change the centre of the plot.
8. Click on reset axis to res-set to default plot
9. “Download plot as a PNG” option can download the plot to the local machine
10. “Autoscale” to adjust the view of the current plot
11. Users can change their internet browser’s size to adjust the view of the whole plot.

[a] McLaren et al. 2016;

[b] <http://www.nealelab.is/uk-biobank>

**
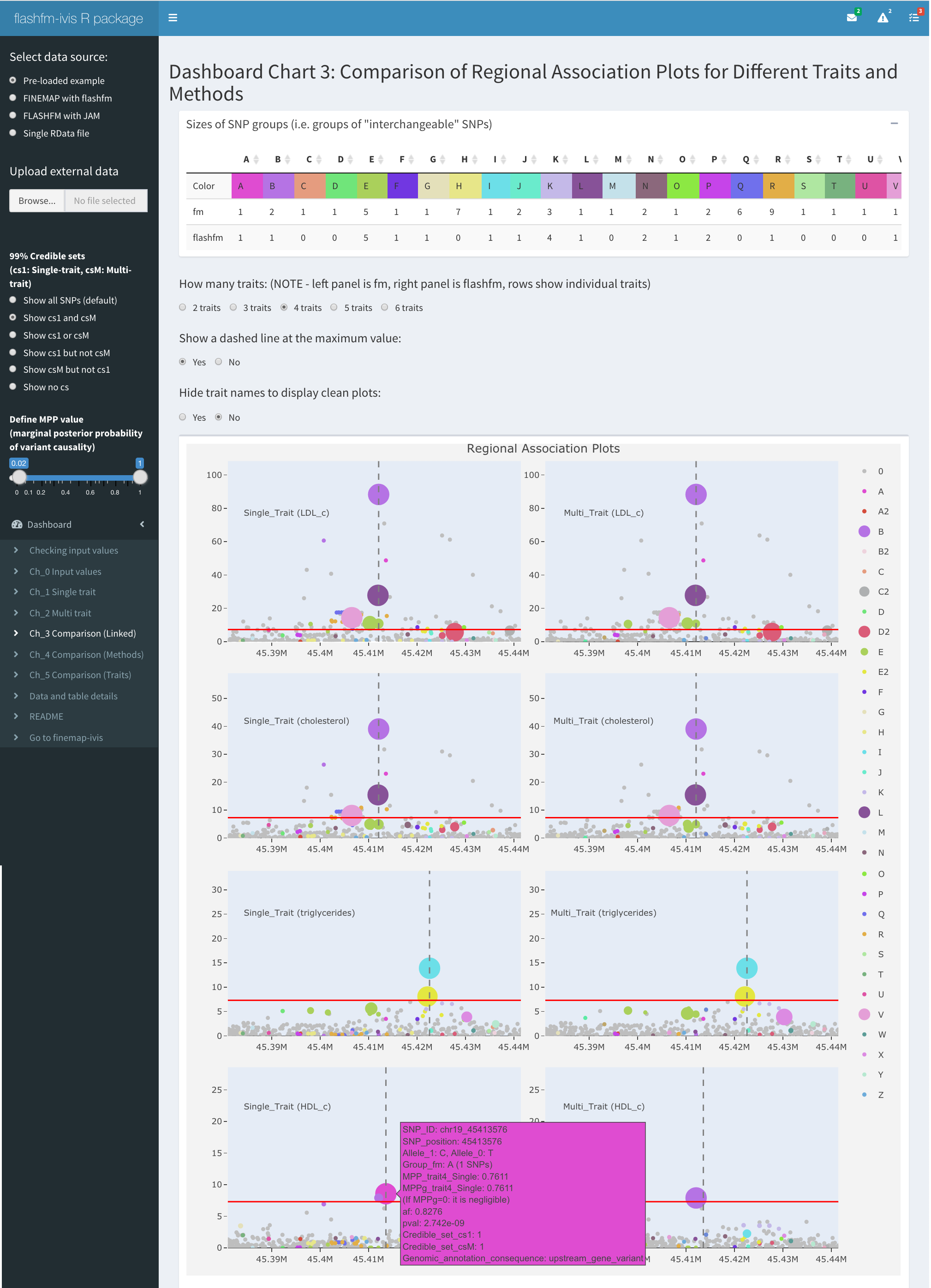
**

*Figure_S8: Ch_3 Comparison (linked)*

**Ch_4 Comparison (Methods) – Compare sizes and SNP overlap of credible sets**

**Radar chart of credible sets – compare sizes of credible sets between traits and methods**

1. Traits appear around the circle and the points indicate the number of SNPs in the fm credible sets (CS_1 is for 1-trait) and the flashfm credible sets (CS_M is for multi-trait)
2. Similar interactive features as other dashboards are available for users to explore the plot

**Area-proportional Venn diagrams - intuitive view of overlap between credible sets**

1. The segments show the number of SNPs that are shared between the intersecting credible sets
2. The area of each segment is proportional to its SNP count

**Interactive** **Venn diagram 1:CS_1 – overlap of fm credible sets between traits**

1. The segments show the number of SNPs that are shared between the intersecting credible sets
2. Hovering over a segment shows the SNP ids belonging to that intersection
3. The downloadable table shows the details of the credible set intersections, such as count and SNP ids. The columns can be sorted by clicking the top row options.

**Interactive Venn diagram 1:CS_M – overlap of flashfm credible sets between traits**

1. The segments show the number of SNPs that are shared between the intersecting credible sets
2. Hovering over a segment shows the SNP ids belonging to that intersection
3. The downloadable table shows the details of the credible set intersections, such as count and SNP ids. The columns can be sorted by clicking the top row options.

**
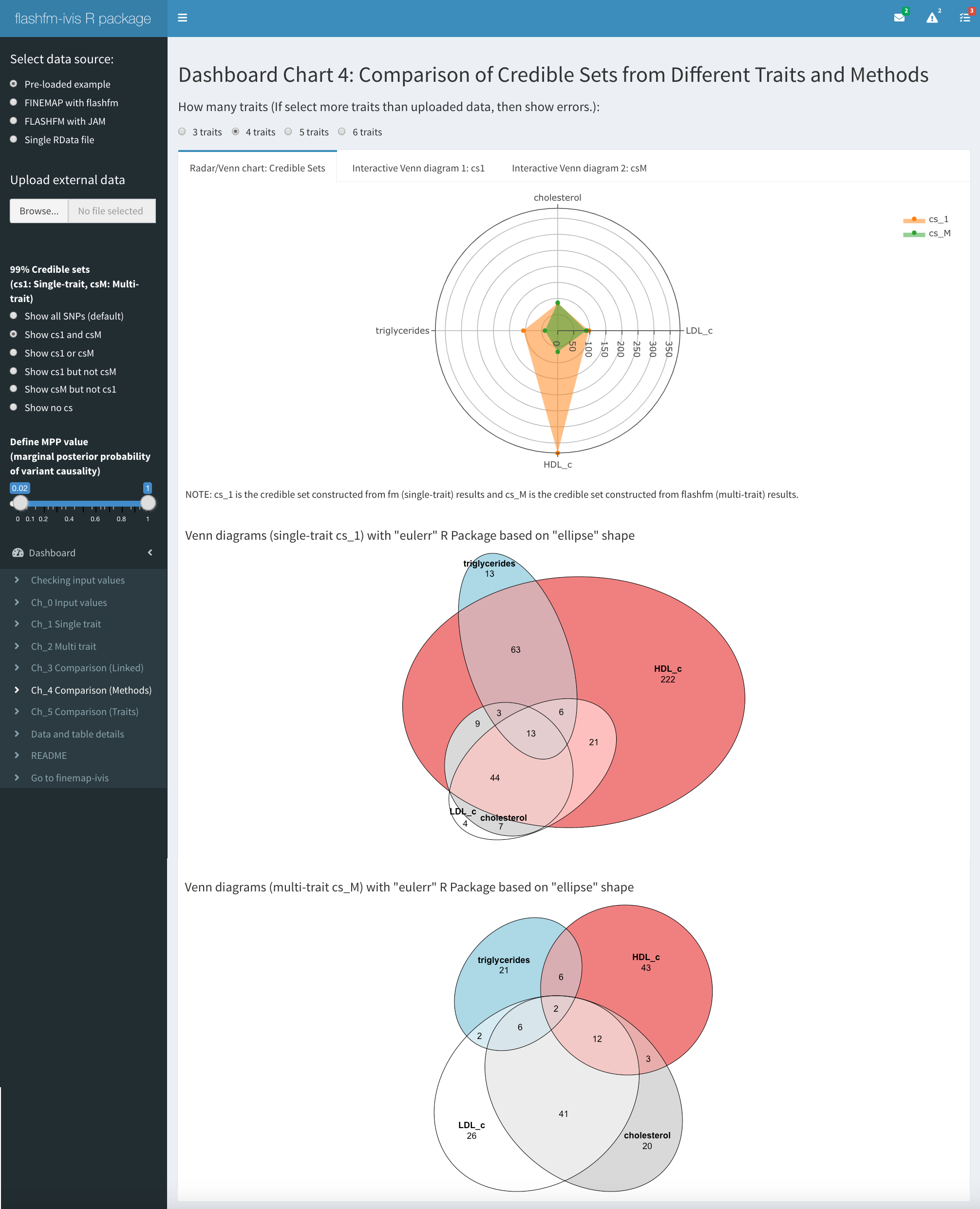
**

*Figure_S9: Ch_4 Comparison (Methods)(a)*

**
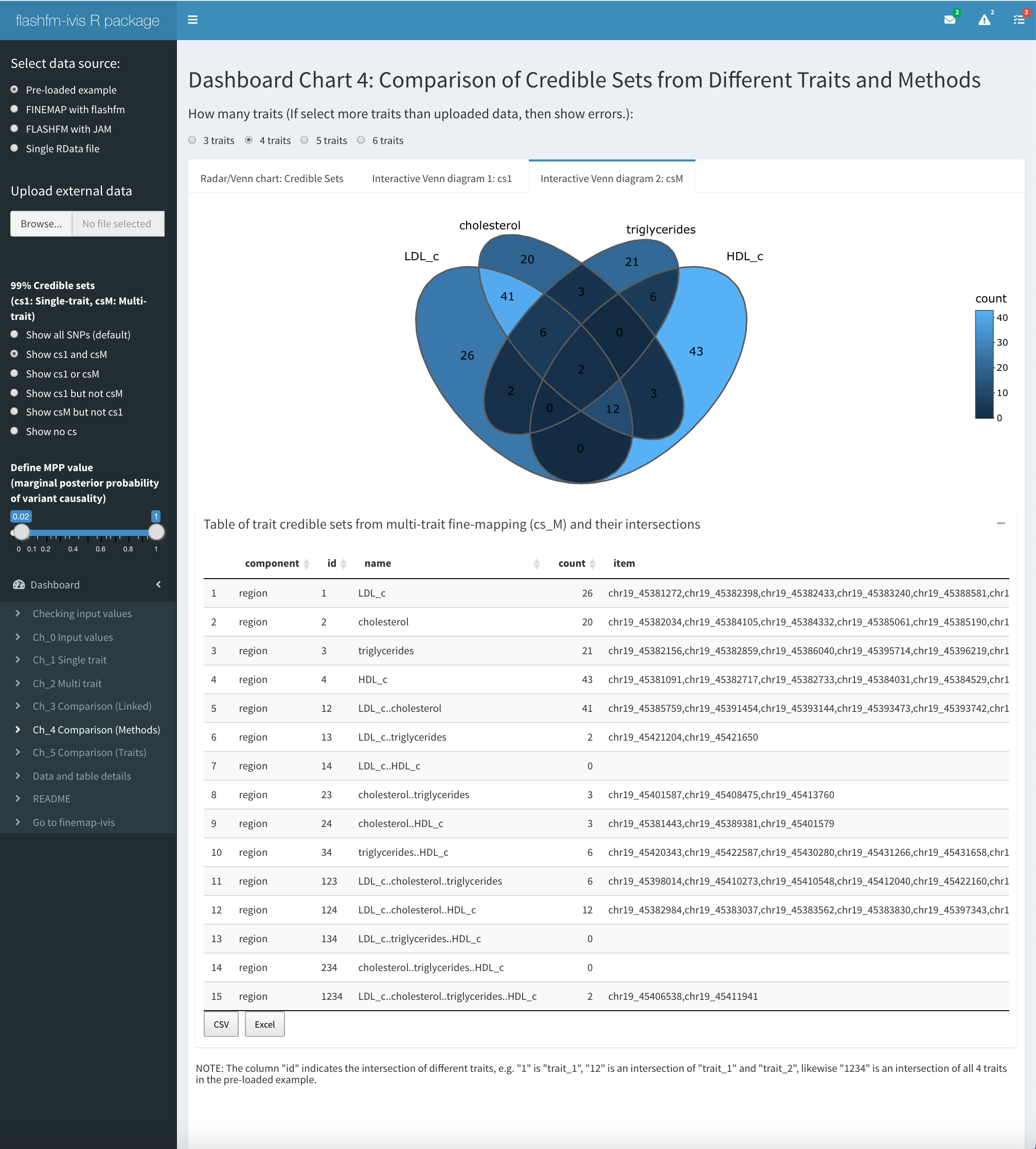
**

*Figure_S10: Ch_4 Comparison (Methods)(b)*

**Ch_5 Comparison (Traits) – Sankey diagrams of traits showing SNP group membership for each method**Each Sankey diagram shows the SNPs that belong to each flashfm SNP group and to each fm SNP group. The flashfm groups tend to be subsets of the fm groups. There is an “All traits” tab and a tab for each trait. For a given fine-mapping method, SNP groups are the same for each trait. Each trait may have different SNP groups that appear in their models. To change the plot perspective (or show the names of SNPs clearly), the positions of the SNPs and groups may be moved by dragging them.

**All traits:** A combined Sankey diagram over all traits. The width of the lines joining the SNPs to their SNP group is proportional to the average MPP (over all traits, including some MPPs that are zero or very close to zero) for the SNP.

**Individual traits:** The width of the lines joining the SNPs to their SNP group is proportional to the trait-specific MPP for the SNP


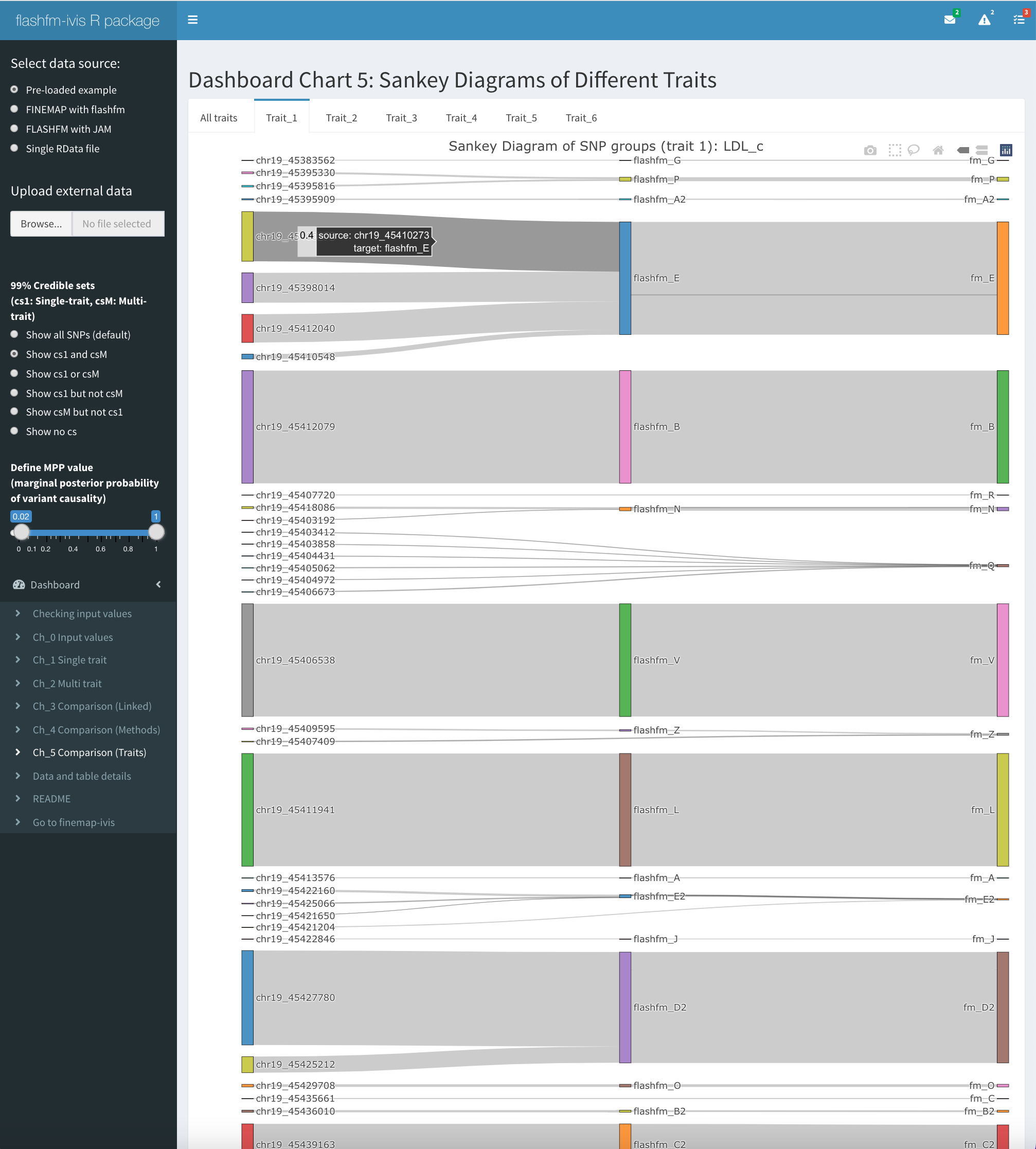


*Figure_S11: Ch_5 Comparison (traits)*

**Data and table details – shows a portion of each input object to verify input**

**
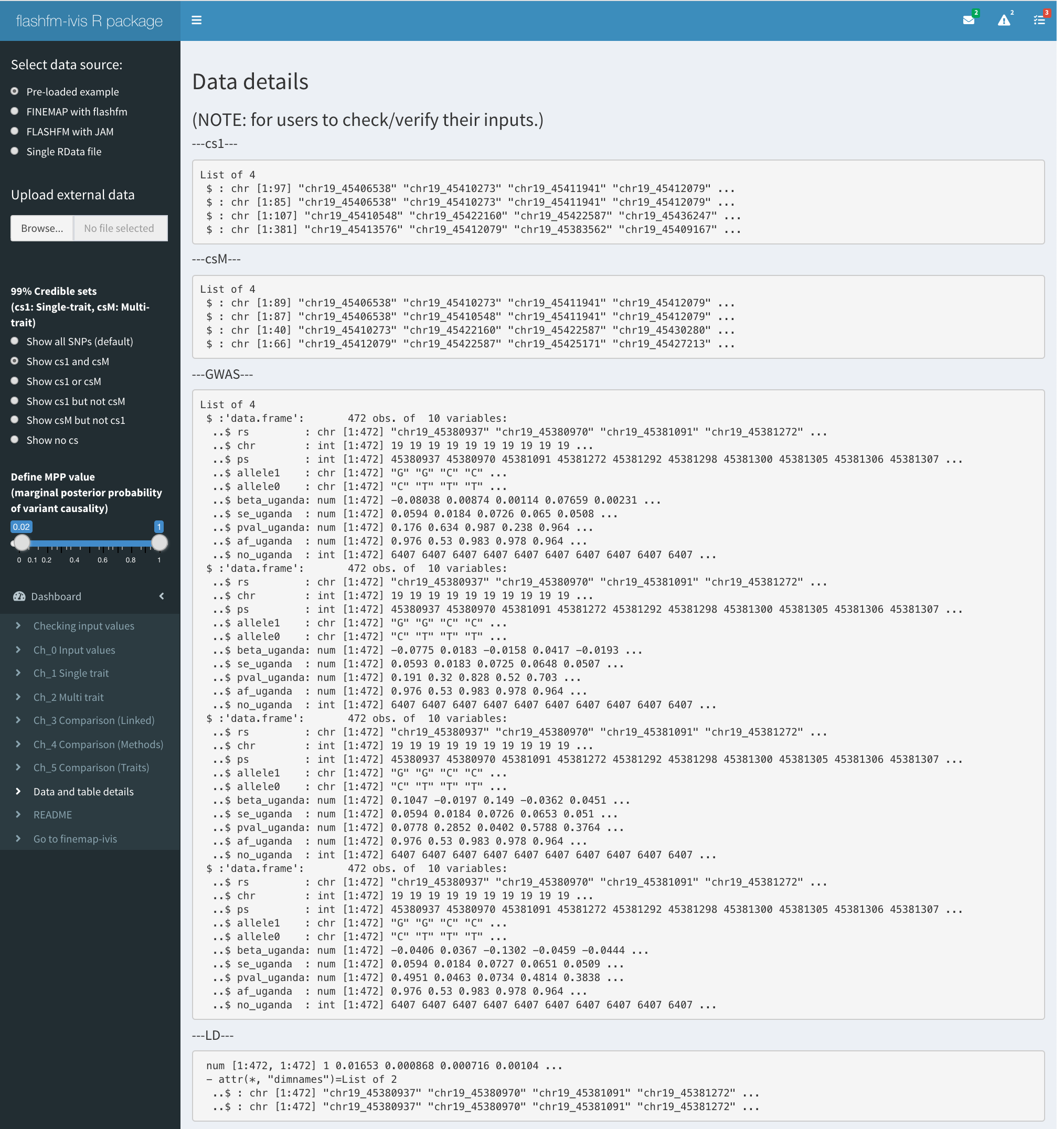
**

*Figure_S12: Data and table details*

**FINEMAP-ivis page:**

**
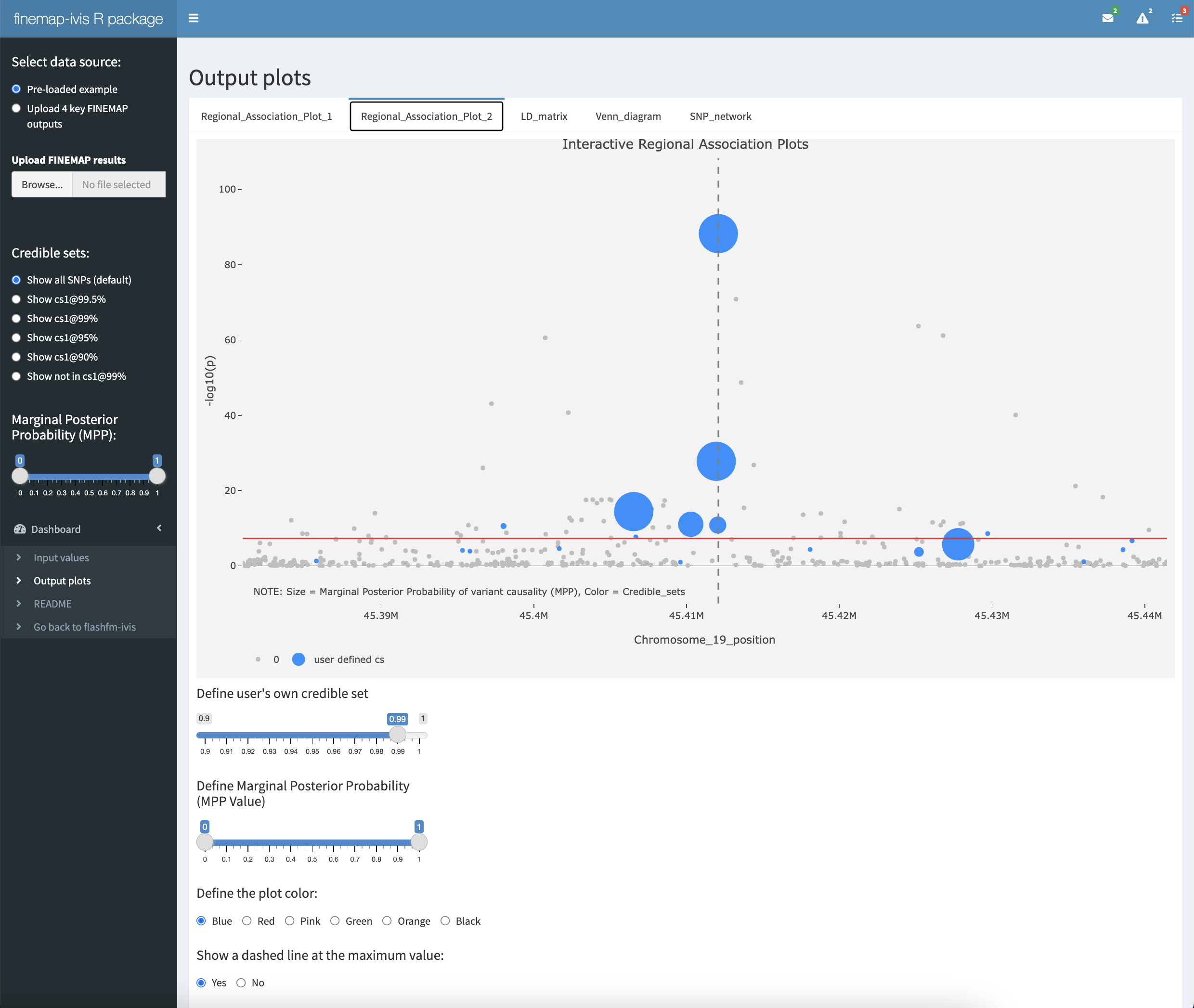
**

*Figure_S13: finemap-ivis*

**References (as included in Supplementary Material)**

1. Allaire, J. J., Ellis, P., Gandrud, C., Kuo, K., Lewis, B. W., Owen, J., ... & Gandrud, M. C. (2017). Package ‘networkD3’. D3 JavaScript Network Graphs from R. <https://christophergandrud.github.io/networkD3/>
2. Barrett, J. C., Fry, B., Maller, J., and Daly, M. J. (2005). Haploview: analysis and visualization of LD and haplotype maps. Bioinformatics 21, 263–265. <https://doi.org/10.1093/bioinformatics/bth457>.
3. Benner, C., Spencer, C. C., Havulinna, A. S., Salomaa, V., Ripatti, S., & Pirinen, M. (2016). FINEMAP: efficient variable selection using summary data from genome-wide association studies. Bioinformatics, 32(10), 1493-1501. <https://doi.org/10.1093/bioinformatics/btw018>.
4. Boughton, A. P., Welch, R. P., Flickinger, M., VandeHaar, P., Taliun, D., Abecasis, G. R., & Boehnke, M. (2021) LocusZoom. js: interactive and embeddable visualization of genetic association study results. *Bioinformatics, 37(18), 3017-3018.* <https://doi.org/10.1093/bioinformatics/btab186>.
5. Buels, R., Yao, E., Diesh, C. M., Hayes, R. D., Munoz-Torres, M., Helt, G., ... & Holmes, I. H. (2016). JBrowse: a dynamic web platform for genome visualization and analysis. Genome biology, 17(1), 1-12. <https://doi.org/10.1186/s13059-016-0924-1>
6. Chang, W (2015) shinydashboard: create dashboards with ‘Shiny’. R package. <https://cran.r-project.org/package=shinydashboard>.
7. Csardi, G., & Nepusz, T. (2006). The igraph software package for complex network research. InterJournal, complex systems, 1695(5), 1-9. <https://igraph.org>
8. Gao, C. H., Yu, G., & Cai, P. (2021). ggVennDiagram: an intuitive, easy-to-use, and highly customizable R package to generate Venn diagram. Frontiers in Genetics, 1598. <https://doi.org/10.3389/fgene.2021.706907>
9. George, G., Gan, S., Huang, Y., Appleby, P., Nar, A. S., Venkatesan, R., ... & Doney, A. S. (2020). PheGWAS: a new dimension to visualize GWAS across multiple phenotypes. Bioinformatics, 36(8), 2500-2505. <https://doi.org/10.1093/bioinformatics/btz944>.
10. He, F., Ding, S., Wang, H., & Qin, F. (2020). IntAssoPlot: An R Package for Integrated Visualization of Genome-Wide Association Study Results With Gene Structure and Linkage Disequilibrium Matrix. Frontiers in genetics, 11, 260. <https://doi.org/10.3389/fgene.2020.00260>
11. Hernandez, N., Soenksen, J., Newcombe, P., Sandhu, M., Barroso, I., Wallace, C., Asimit, J.L. (2021). The flashfm approach for fine-mapping multiple quantitative traits. *Nat Commun* 12, 6147. <https://doi.org/10.1038/s41467-021-26364-y>
12. Hu, B., Jin, J., Guo, A., Zhang, H., Luo, J., and Gao, G. (2015). GSDS 2.0: an upgraded gene feature visualization server. Bioinformatics 31, 1296–1297. <https://doi.org/10.1093/bioinformatics/btu817>.
13. Khramtsova, E. A., & Stranger, B. E. (2017). Assocplots: a Python package for static and interactive visualization of multiple-group GWAS results. Bioinformatics, 33(3), 432-434. <https://doi.org/10.1093/bioinformatics/btw641>.
14. Kichaev, G., Roytman, M., Johnson, R., Eskin, E., Lindstroem, S., Kraft, P., & Pasaniuc, B. (2017). Improved methods for multi-trait fine mapping of pleiotropic risk loci. Bioinformatics, 33(2), 248-255. <https://doi.org/10.1093/bioinformatics/btw615>.
15. Kierczak, M., Jablonska, J., Forsberg, S. K., Bianchi, M., Tengvall, K., Pettersson, M., et al. (2015). Cgmisc: enhanced genome-wide association analyses and visualization. Bioinformatics 31, 3830–3831. <https://doi.org/10.1093/bioinformatics/btv426>.
16. Kortemeier,E. et al. (2018) ShinyGPA: an interactive visualization toolkit for investigating pleiotropic architecture using GWAS datasets. PLoS One, 13, e0190949. https://doi.org/10.1371/journal.pone.0190949
17. Kwong, A., Boughton, A. P., Wang, M., VandeHaar, P., Boehnke, M., Abecasis, G., & Kang, H. M. (2021). FIVEx: an interactive eQTL browser across public datasets. Bioinformatics. <https://doi.org/10.1093/bioinformatics/btab614>.
18. Lipka, A. E., Tian, F., Wang, Q., Peiffer, J., Li, M., Bradbury, P. J., et al. (2012). GAPIT: genome association and prediction integrated tool. Bioinformatics 28, 2397–2399. <https://doi.org/10.1093/bioinformatics/bts444>.
19. Machiela, M. J., and Chanock, S. J. (2015). LDlink: a web-based application for exploring population-specific haplotype structure and linking correlated alleles of possible functional variants. Bioinformatics 31, 3555–3557. <https://doi.org/10.1093/bioinformatics/btv402>.
20. McLaren W, Gil L, Hunt SE, Riat HS, Ritchie GR, Thormann A, Flicek P, Cunningham F. (2016). The Ensembl Variant Effect Predictor. Genome Biology Jun 6;17(1):122. [doi:10.1186/s13059-016-0974-4](https://genomebiology.biomedcentral.com/articles/10.1186/s13059-016-0974-4)
21. Newcombe, P. J., Conti, D. V. & Richardson, S. (2016). JAM: a scalable Bayesian framework for joint analysis of marginal SNP effects. *Genet. Epidemiol.* 40, 188–201. <https://onlinelibrary.wiley.com/doi/full/10.1002/gepi.21953>
22. Pruim, R. J., Welch, R. P., Sanna, S., Teslovich, T. M., Chines, P. S., Gliedt, T. P., ... & Willer, C. J. (2010). LocusZoom: regional visualization of genome-wide association scan results. Bioinformatics, 26(18), 2336-2337. <https://doi.org/10.1093/bioinformatics/btq419>.
23. Schilder, B. M., Humphrey, J., & Raj, T. (2021). echolocatoR: an automated end-to-end statistical and functional genomic fine-mapping pipeline. Bioinformatics. 1-4, <https://doi.org/10.1093/bioinformatics/btab658>.
24. Sievert, C. (2020). Interactive web-based data visualization with R, plotly, and shiny. CRC Press.
25. Verity, R., Collins, C., Card, D. C., Schaal, S. M., Wang, L., & Lotterhos, K. E. (2017). minotaur: A platform for the analysis and visualization of multivariate results from genome scans with R Shiny. Molecular ecology resources, 17(1), 33-43. <https://doi.org/10.1111/1755-0998.12579>
26. Wang J., Zhang Z. (2021). GAPIT Version 3: Boosting Power and Accuracy for Genomic Association and Prediction, Genomics, Proteomics & Bioinformatics, doi: <https://doi.org/10.1016/j.gpb.2021.08.005>.
27. Wickham, H. (2011). ggplot2. Wiley interdisciplinary reviews: computational statistics, 3(2), 180-185.
28. Ziegler,G.R. et al. (2015) Zbrowse: an interactive GWAS results browser. PeerJ Comput. Sci., 1, e3. https://doi.org/10.7287/peerj.preprints.902v1
